# Temporal behavior of proportional mortality of fetal deaths according to underlying cause, 2011–2020

**DOI:** 10.1590/1980-220X-REEUSP-2025-0288en

**Published:** 2026-02-16

**Authors:** Júlia Rodrigues Soares de Barros, Ana Beatriz Henrique Parenti, Ana Paula Pinho Carvalheira, Anna Paula Ferrari, Marli Teresinha Cassamassimo Duarte, Cristina Maria Garcia de Lima Parada

**Affiliations:** 1 Universidade Estadual Paulista “Júlio de Mesquita Filho”, Faculdade de Medicina de Botucatu, Departamento de Enfermagem, Botucatu, SP, Brazil.

**Keywords:** Fetal Death, Epidemiology, Risk Factors, Health Information Systems

## Abstract

**Objective:**

To investigate the temporal behavior of proportional mortality of early, intermediate, and late fetal deaths between 2011 and 2020, according to underlying cause.

**Methods:**

Population-based study conducted with secondary data on fetal deaths recorded in the Mortality Information System for the period between 2011 and 2020.

**Results:**

The highest annual averages of proportional mortality according to the underlying cause of early, intermediate, or late fetal deaths were, respectively: fetus and newborn affected by maternal factors and complications of pregnancy, labor, and delivery (41.3%, 44.4%, and 44.0%); intrauterine hypoxia and asphyxia at birth (22.3%, 21.6%, and 22.8%); and other conditions originating in the perinatal period (28.0%, 23.4%, and 24.4%). Hypoxia and asphyxia showed a downward trend for all types of death. The group of fetuses and newborns affected by maternal factors and complications of pregnancy, labor, and delivery showed an increasing trend.

**Conclusion:**

The main underlying causes of death are characterized as preventable through adequate prenatal, delivery, and newborn care, which highlights the need to improve perinatal care in order to reduce and/or prevent their occurrence.

## INTRODUCTION

The World Health Organization (WHO) and the International Statistical Classification of Diseases and Related Health Problems – 11th revision (ICD-11) define fetal death as the death of a fetus before its complete expulsion or extraction from a woman’s body, regardless of the duration of pregnancy^(1)^. It is a significant global public health problem and an important indicator of maternal and child health^(2)^, as it reflects the quality of care and access to healthcare available to pregnant women from prenatal care to the moment of delivery^(3,4)^.

Fetal death can be classified as early (less than 20 weeks of gestation), intermediate (between 20 and 28 weeks), and late (after 28 weeks), or as antepartum or intrapartum, according to the period of occurrence^(5)^, with this negative outcome being more frequent in middle- and low-income countries^(6)^. In high-income countries, such as the United States of America, improvements in care for pregnant women over the last century have resulted in a significant reduction in fetal deaths and a proportionally greater decline in neonatal deaths, so that fetal death remains a significant and understudied problem, accounting for almost 50% of all perinatal deaths in the country^(7)^. In Brazil, the overall fetal mortality rate between 1996 and 2021 was 11.4/1,000 live births, with all regions showing a downward trend, with some occasional fluctuations, and the sharpest decline recorded in the Southeast region, with a 48% reduction^(8)^.

Fetal death can be influenced by the simultaneous occurrence of several factors, and this multifactorial nature can make it difficult to detect the specific cause^(9)^. Factors associated with adverse perinatal outcomes, including fetal death, include: maternal age over 35 years; nulliparity; smoking; previous fetal losses; previous maternal diseases, such as hypertension, diabetes mellitus, and obesity; maternal infections during pregnancy; placental, umbilical, amniotic, or uterine complications; congenital anomalies; asphyxia and birth trauma; fetal growth restriction; and absence or poor quality of prenatal care. In addition, some social factors, such as low educational attainment, black or brown maternal skin color, and poor socioeconomic conditions are also associated with fetal deaths^(10)^.

A national study on fetal deaths between 1996 and 2021^(8)^ presented them based on the overall mortality rate, without considering the classification according to gestational age at the time of occurrence, a gap that this study aims to address. The study investigates the temporal behavior of proportional mortality of early, intermediate, and late fetal deaths between 2011 and 2020, according to their underlying cause and macro-region of occurrence, with a view to supporting actions to improve maternal and child health and the adoption of preventive measures to avoid their occurrence.

## METHOD

### Study Design

This is an observational, retrospective, population-based study based on secondary data from the Mortality Information System (SIM in the Portuguese acronym) on fetal deaths. The STROBE Declaration was used in its construction^(11)^.

### Population and Data Source

All cases of fetal death that occurred in Brazil and were recorded in the SIM between 2011 and 2020 were included in the study. The SIM is a national epidemiological surveillance system that aims to collect data on deaths in the country. Its basic document is the Death Certificate (DC), which is mandatory, so that the mortality statistics produced are valid and comparable. The system has a completeness rating classified as excellent (less than 5% incompleteness) and, despite disparities in the quality of its different dimensions, studies indicate improvements over time in the recording of mortality data for the Brazilian population^(12)^.

### Study Variables

Variables related to maternal and fetal characteristics, pregnancy and childbirth data, and death were used: Maternal: age (full years); education (up to 3, 4–7, 8–11, 12 or more); paid activity (yes, no); number of living children (0–3, ≥4); dead children (yes, no); region of residence (North, Northeast, Center West, Southeast, South). Fetal: sex (male, female); birth weight (<1500, 1500–2499, ≥2500); race/skin color (White, Brown, Black, Yellow, Indigenous). Pregnancy and delivery data: type of pregnancy (single, multiple); type of delivery (vaginal, cesarean); gestational age (full weeks). Data related to death: trimester of occurrence (first, second, and third); time of death (before delivery, after delivery); macro-region of occurrence (North, Northeast, Center West, Southeast, South); medical assistance at death (yes, no). Underlying cause by group, according to ICD- 10 chapter^(13)^: Chapters: I - infectious and parasitic diseases; II - neoplasms/tumors; III - diseases of the blood, hematopoietic organs, and some immune disorders; IV - endocrine, nutritional, and metabolic diseases; V - mental and behavioral disorders; VI - diseases of the nervous system; VII - diseases of the eye and adnexa; VIII - diseases of the ear and mastoid process; IX - diseases of the circulatory system; X - diseases of the respiratory system; XI - diseases of the digestive system; XII - diseases of the skin and subcutaneous tissue; XIII - diseases of the musculoskeletal system and connective tissue; XIV - diseases of the genitourinary system; XV - pregnancy, childbirth, and the puerperium; XVI - certain conditions originating in the perinatal period; XVII - congenital malformations, deformities, and chromosomal abnormalities; XVIII - symptoms, signs, and abnormal clinical and laboratory findings, not elsewhere classified; XIX - injuries, poisoning, and certain other consequences of external causes; XX - external causes of morbidity and mortality; XXI - factors influencing health status and contact with health services; XXII - codes for special purposes.

### Statistical Methods

Initially, a descriptive analysis was performed of the variables related to maternal and fetal characteristics, pregnancy, childbirth, and death. To investigate the existence of a trend toward an increase or decrease in the proportional mortality of fetal deaths by underlying cause and macro-region of occurrence in the populations of early, intermediate, and late fetal deaths, linear regression models with normal response were adjusted for each time series of proportional mortality, considering time as an independent variable. In addition, the trend for each series was tested using the Cox-Stuart test. Analyses were performed using SPSS 21 software.

### Ethical Aspects

Ethical aspects were ensured in accordance with Resolution No. 510 of the National Health Council, dated April 7, 2016, sole paragraph, which exempts research using publicly available information from registration and evaluation by the Research Ethics Committee/National Research Ethics Commission (CEP/CONEP) system^(14)^.

## RESULTS

In the decade 2011-2020, there were 164.504 fetal deaths registered in the SIM. Chapter 16 of ICD-10 (certain conditions originating in the perinatal period) included most of them, followed by Chapter 17 (congenital malformations, deformities, and chromosomal abnormalities) and Chapter 1 (certain infectious and parasitic diseases), as shown in Table 1.

**Table 1:** Description of deaths by ICD-10 chapter – Brazil, 2011–2020.

Chapter	Category	n	%
**1 infectious and parasitic diseases**		
Congenital syphilis		2340	1.4
**16 some conditions originating in the perinatal period**			
Fetus and newborn affected by maternal factors and complications of pregnancy, labor and delivery		72436	44.0
Other conditions originating in the perinatal period		40138	24.4
Intrauterine hypoxia and birth asphyxia		36891	22.4
Fetal growth restriction, fetal malnutrition and disorders related to short gestation and low birth weight		1844	1.1
Hemolytic disease of the fetus and newborn		266	0.2
Other infections specific to the perinatal period		277	0.2
Congenital infectious and parasitic diseases		175	0.1
Trauma during birth		98	0.06
Other respiratory disorders originating in the perinatal period		48	0.03
**17 congenital malformations, deformities and chromosomal anomalies**		3997	2.4
Other congenital malformations			
Other congenital malformations of the nervous system		2409	1.5
Chromosomal abnormalities, not elsewhere classified		1049	0.7
Congenital malformations of the circulatory system		1247	0.7
Other congenital malformations and deformities of the musculoskeletal system		662	0.4
Other malformations of the genitourinary system		377	0.2
Other congenital malformations of the digestive system		129	0.08
Spina bifida		74	0.04
Cleft lip and cleft palate		31	0.02
Absence, atresia and stenosis of the small intestine		16	0.01

Regarding the annual averages of proportional mortality, according to underlying cause, the following categories stood out, considering the occurrence of early, intermediate, or late fetal death, respectively: fetus and newborn affected by maternal factors and complications of pregnancy, labor, and delivery (41.3%, 44.4%, and 44.0%); intrauterine hypoxia and asphyxia at birth (22.3%, 21.6%, and 22.8%); and other conditions originating in the perinatal period (28.0%, 23.4%, and 24.4%), all from Chapter 16 of ICD-10 (data not shown in table).

Intrauterine hypoxia and asphyxia at birth tended to decrease for all types of death: early, intermediate, and late. There was also a downward trend for other specific perinatal infections in cases of intermediate fetal deaths and other malformations of the nervous system among late fetal deaths (Table 2).

**Table 2:** Investigation of the trend in proportional fetal mortality according to underlying cause of death – Brazil, 2011–2020.

Category	Early			Intermediate			Late		
	β (95%CI)	*p*	Trend	β (95%CI)	*p*	Trend	β (95%CI)	*p*	Trend
Chromosomal abnormalities, not elsewhere classified	0.05 (0.01–0.09)	*0.019*	Increase	0.05 (0.03–0.08)	*0.001*	Increase	0.06 (0.04–0.07)	*<0.001*	Increase
Absence, atresia and stenosis of the small intestine	0.00 (–0.01–0.01)	*0.873*	Stationary	0.00 (0.00–0.00)	*0.244*	Stationary	0.00 (0.00–0.00)	*0.700*	Stationary
Hemolytic disease of the fetus and newborn	0.00 (–0.02–0.02)	*0.752*	Stationary	0.01 (–0.01–0.02)	*0.223*	Stationary	0.00 (–0.01–0.00)	*0.194*	Stationary
Congenital infectious and parasitic diseases	–0.02 (–0.04–0.00)	*0.074*	Stationary	0.00 (–0.01–0.01)	*0.778*	Stationary	0.00 (0.00–0.01)	*0.168*	Stationary
Spina bifida	0.01 (–0.01–0.02)	*0.388*	Stationary	0.01 (–0.01–0.02)	*0.287*	Stationary	0.00 (–0.01–0.00)	*0.274*	Stationary
Cleft lip and cleft palate	0.00 (–0.01–0.00)	*0.122*	Stationary	0.00 (0.00–0.00)	*0.997*	Stationary	0.00 (0.00–0.00)	*0.491*	Stationary
Fetus and newborn affected by maternal factors and complications of pregnancy, labor and delivery	0.74 (0.26–1.21)	*0.007*	Increase	0.53 (0.27–0.78)	*0.001*	Increase	0.53 (0.28–0.79)	*0.001*	Increase
Intrauterine hypoxia and birth asphyxia	–0.44 (–0.81– –0.07)	*0.024*	Decrease	–0.48 (–0.67– –0.28)	*<0.001*	Decrease	–0.55 (–0.84– –0.26)	*0.002*	Decrease
Congenital malformations of the circulatory system	0.01 (–0.04–0.06)	*0.615*	Stationary	0.05 (0.02–0.07)	*0.004*	Increase	0.04 (0.02–0.07)	*0.002*	Increase
Other conditions originating in the perinatal period	–0.31 (–0.92–0.30)	*0.273*	Stationary	–0.19 (–0.47–0.09)	*0.151*	Stationary	–0.19 (–0.67–0.29)	*0.394*	Stationary
Other infections specific to the perinatal period	0.02 (–0.02–0.05)	*0.340*	Stationary	–0.02 (–0.03– –0.01)	*0.002*	Decrease	–0.01 (–0.02–0.00)	*0.077*	Stationary
Other congenital malformations	–0.03 (–0.11–0.05)	*0.360*	Stationary	0.04(–0.06– 0.13)	*0.401*	Stationary	0.00 (–0.04–0.03)	*0.889*	Stationary
Other congenital malformations of the digestive system	0.00 (–0.01–0.01)	*0.737*	Stationary	0.00 (–0.01–0.01)	*0.550*	Stationary	0.00 (–0.01–0.00)	*0.035*	Not relevant
Other congenital malformations of the nervous system	0.05 (–0.01–0.11)	*0.107*	Stationary	0.02 (–0.06–0.09)	*0.613*	Stationary	–0.05 (–0.08– –0.03)	*0.002*	Decrease
Other malformations of the Genitourinary	0.02 (–0.02–0.07)	*0.278*	Stationary	0.00 (–0.03–0.03)	*0.913*	Stationary	0.01 (0.00–0.03)	*0.089*	Stationary
Other malformations and congenital deformities of the musculoskeletal system	0.03 (0.00–0.06)	*0.038*	Enlargement	0.01 (–0.01–0.02)	*0.535*	Stationary	0.02 (0.00–0.03)	*0.027*	Increase
Other respiratory disorders originating in the perinatal period	0.00 (–0.01–0.01)	*0.742*	Stationary	0.00 (–0.01–0.01)	*0.633*	Stationary	0.00 (–0.01–0.00)	*0.794*	Stationary
Fetal growth retardation, fetal malnutrition and disorders related to short gestation and low birth weight	–0.15 (–0.36–0.06)	*0.127*	Stationary	–0.04 (–0.18–0.09)	*0.479*	Stationary	0.03 (0.00–0.06)	*0.085*	Stationary
Congenital syphilis	0.03 (–0.03–0.08)	*0.345*	Stationary	0.04 (–0.06–0.15)	*0.400*	Stationary	0.13 (0.06–0.19)	*0.003*	Increase
Trauma during birth	0.00 (–0.01–0.02)	*0.721*	Stationary	–0.01 (–0.02–0.00)	*0.119*	Stationary	0.00 (–0.01–0.01)	*0.295*	Stationary

The categories that showed a statistically significant increase in the trend for all types of death were: chromosomal abnormalities not classified elsewhere and fetus and newborn affected by maternal factors and complications of pregnancy, labor, and delivery. There was a tendency toward an increase in the case of early fetal deaths for other malformations and congenital musculoskeletal deformities. For intermediate and late deaths, there was an increasing trend for congenital malformations of the circulatory system, and only among late deaths was there still an increasing trend for other congenital malformations and deformities of the musculoskeletal system and congenital syphilis (Table 2).

**Figure 1:**
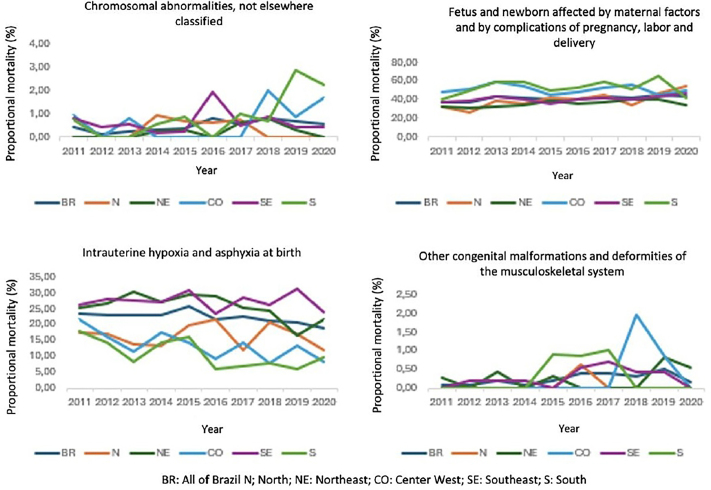
Analysis of the annual trend in early fetal deaths by macro-region of occurrence and ICD-10 category.

Among early fetal deaths, the time series of proportional mortality according to the categories: chromosomal anomalies not classified elsewhere; fetus and newborn affected by maternal factors and complications of pregnancy, labor, and delivery, and other congenital malformations and deformities of the musculoskeletal system showed an annual upward trend, while the series of proportional mortality according to the category intrauterine hypoxia and birth asphyxia showed an annual downward trend (Figure 1).

With regard to the proportional mortality of early fetal deaths, according to macro-regions, an increase was observed in the following categories: chromosomal abnormalities in the South region (β = 0.23, 95% CI = 0.04–0.41, p = 0.021); fetus and newborn affected by maternal factors and complications of pregnancy, labor, and delivery in the North (β = 2.10, 95% CI = 0.82–3.38, p = 0.005) and a downward trend in the category of intrauterine hypoxia and asphyxia at birth in the Central West and South, respectively (β = –1.05, 95% CI = –1.84 – –0.25, p = 0.017) and (β = –1.00, 95% CI = –1.89 – –0.11, p = 0.032). There were no significant results in the category of other malformations and congenital deformities of the musculoskeletal system by macro-region (Figure 1).

**Figure 2:**
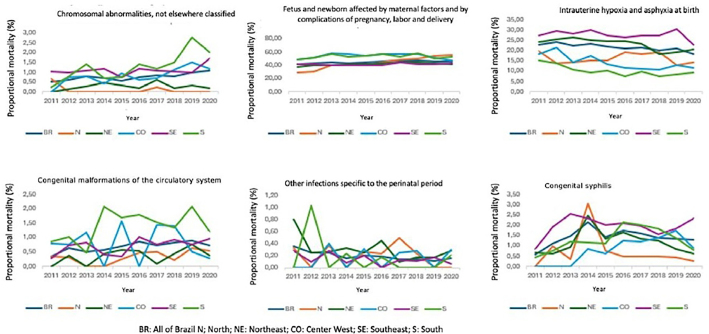
Analysis of the annual trend in intermediate fetal deaths by macro-region of occurrence and ICD-10 category.

Among intermediate fetal deaths, the time series of proportional mortality according to the categories: chromosomal abnormalities not classified elsewhere; fetus and newborn affected by maternal factors and complications of pregnancy, labor, and delivery, and congenital malformations of the circulatory system increased, while the categories intrauterine hypoxia and asphyxia at birth and other specific infections of the perinatal period decreased in the same period (Figure 2).

Regarding the proportional mortality of intermediate fetal deaths, according to macro-regions, an increase was observed in the categories: chromosomal abnormalities in the South and Center West regions (β = 0.20, 95% CI = 0.08–0.31, p = 0.004) and (β = 0.11, 95% CI = 0.004 –0.18, p = 0.006), respectively; fetus and newborn affected by maternal factors and complications of pregnancy, labor, and delivery in the Northern (β = 2.88, 95% CI = 2.34–3.42, p < 0.001) and Northeast (β = 0.50, 95% CI = 0.21–0.79, p = 0.004) and congenital syphilis in the Center West region (β = 0.17, 95% CI = 0.08–0.27, p = 0.002). There was a downward trend in the categories of intrauterine hypoxia and birth asphyxia in the Northeast, Center West, and South (β = –0.77, 95% CI= –1.21– –0.33, p = 0.004), (β = –0.95, 95% CI= –1.52 – –0.38, p = 0.005) and (β = –0.67, 95% CI = –1.08 – –0.25, p = 0.006), respectively. There were no significant results in the category of other specific perinatal infections and congenital malformations of the circulatory system when considering the macro-region of occurrence (Figure 2).

**Figure 3:**
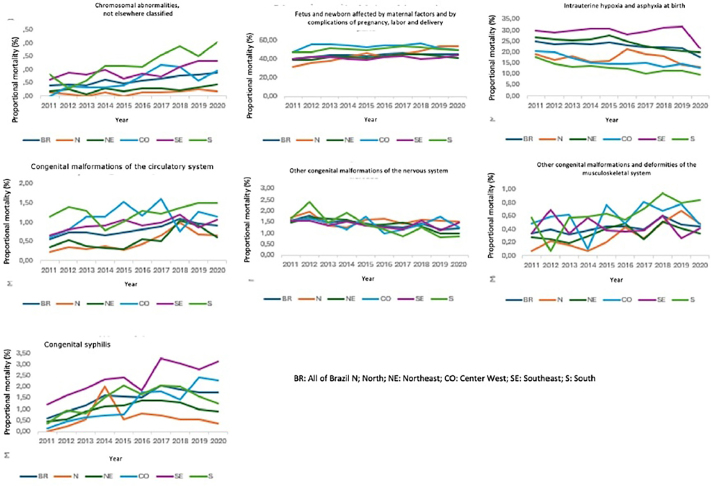
Analysis of the annual trend in late fetal deaths by macro-region of occurrence and ICD-10 category.

Among late fetal deaths, the time series of proportional mortality according to the categories: chromosomal anomalies not classified elsewhere; fetus and newborn affected by maternal factors and complications of pregnancy, labor, and delivery; congenital malformations of the circulatory system; other congenital malformations and deformities of the musculoskeletal system, and congenital syphilis of ICD-10 showed an annual upward trend, while the series of proportional mortality according to the category: intrauterine hypoxia and asphyxia at birth and other congenital malformations of the nervous system showed an annual downward trend (Figure 3).

Regarding the proportional mortality of late fetal deaths, according to macro-regions, an increase was observed in the categories of chromosomal abnormalities in the Northeast (β = 0.02, 95% CI = 0.00–0.04, p = 0.036), Center West (β = 0.10, 95% CI = 0.04–0.17, p = 0.0050, Southeast (β = 0.06, 95% CI = 0.02–0.11, p = 0.010), and South (β = 0.17, 95% CI = 0.10–0.23, p < 0.001); fetuses and newborns affected by maternal factors and complications of pregnancy, labor, and delivery in the North (β = 2.35, 95% CI = 1.83–2.87, p < 0.001) and Northeast (β = 0.42, 95% CI = 0.00–0.84, p = 0.005); malformation of the circulatory system in the North (β = 0.06, 95% CI = 0.02–0.10, p = 0.005), Northeast (β = 0.06, 95% CI = 0.01–0.11, p = 0.035), and Southeast (β = 0.03, 95% CI = 0.00–0.06, p = 0.027); nervous system malformation in the Northeast (β = –0.07, 95% CI= –0.11 – –0.03, p = 0.002) and South (β = –0.14, 95% CI= –0.22 – –0.06, p = 0.004); musculoskeletal malformation in the Northern (β = 0.06, 95% CI = 0.03–0.09, p = 0.002) and Southern (β = 0.06, 95% CI = 0.01–0.10, p = 0.014) regions and syphilis in the Center West (β = 0.25, 95% CI = 0.18–0.32, p < 0.001) and Southeast (β = 0.20, 95% CI = 0.11–0.29, p = 0.001) regions. There was a downward trend in the categories of intrauterine hypoxia and birth asphyxia in the Northeast (β =.–0.82, 95% CI = –1.18 – –0.46, p = 0.001), Center West (β = –0.79, 95% CI = –1.10 – –0.47, p < 0.001), and South (β= –0.70, 95% CI = –0.97 – –0.43, p < 0.001) (Figure 3).

## DISCUSSION

This study allowed us to understand the temporal behavior of proportional mortality of fetal deaths according to underlying cause in the period from 2011 to 2020, with chapter 16 of ICD-10 (some conditions originating in the perinatal period) including most of the fetal deaths that occurred: 92.5%, followed by chapter 17 (congenital malformations, deformities, and chromosomal abnormalities): 6.1% and chapter 1 (infectious and parasitic diseases): 1.4%.

Based on the above, it was possible to identify an upward trend in the underlying cause of fetal and newborn death affected by maternal factors and complications of pregnancy, labor, and delivery in the North and Northeast regions. Regarding the other causes, it was not possible to identify a pattern of increase or decrease in the trend of proportional fetal mortality according to the underlying cause of death in the different regions of the country.

A study that analyzed fetal deaths in the Center West region between 2008 and 2018 showed that 93% of the deaths included in the study were associated with chapter 16 of the ICD-10 (some conditions originating in the perinatal period), a finding similar to that of the present study, where 92.5% of deaths were also associated with chapter 16. It should be noted that the vast majority of conditions originating in the perinatal period are preventable, suggesting the need for improvement in the quality of care provided, both prenatally and at birth^(15,16)^.

Perinatal asphyxia, which includes intrauterine hypoxia and birth asphyxia, showed a downward trend for all types of deaths. A study that analyzed the epidemiology of perinatal mortality, according to the Wigglesworth classification, highlighted that, of the 58.9% of fetal deaths, only 0.8% were caused by asphyxia^(17)^. The relevance of this category in the present study stems from the high frequency identified in the period, since approximately 20% of deaths had this underlying cause. Given this, the result of the downward trend in the occurrence of early, intermediate, and late fetal deaths related to this cause indicates that the quality of care provided to both the pregnant woman and the fetus may have improved over the study period. A study that aimed to analyze indicators related to prenatal care, using information from the 2013 and 2019 editions of the National Health Survey, found a significant increase in the number of prenatal consultations and clinical-obstetric examinations, in the proportion of women who received guidance on referral services for childbirth, and in the performance of tests for syphilis and HIV. The authors concluded that there have been important advances in the coverage and quality of care for pregnant women in Brazil, although challenges remain, such as late initiation of prenatal care and insufficient guidance on referral services for childbirth^(18)^.

The decrease in the occurrence of fetal deaths due to nervous system malformations may be due to the introduction of folic acid supplementation in the periconceptional period and during the first trimester of pregnancy, which resulted in a decrease of approximately 50% in neural tube defects, as well as the early diagnosis of malformations from imaging tests performed during pregnancy and new intrauterine neurosurgical techniques, improving perinatal outcomes^(19)^.

With regard to chromosomal abnormalities not classified elsewhere, a study conducted in French Guiana highlighted that 6% of deaths were due to fetal anomalies, with almost 50% of these deaths resulting from chromosomal anomalies^(20)^. A study conducted in Northeastern Brazil showed that, although 2.9% of fetal deaths occurred due to congenital malformations, there was a statistically significant increase in these cases^(21)^. A population-based ecological study conducted in Brazil compared malformations among women aged 35–40 and over 41 and, contrary to expectations, when malformations were analyzed by age subgroup, there was a tendency for an increase only among younger women. The authors pointed out that this finding may be due to the small size of the older subgroup^(22)^.

As there is evidence of an association between malformation and advanced maternal age, one of the factors that may be related to the aforementioned increase is women’s choice to delay pregnancy, resulting from the urbanization process and their interest in education, factors related to the decline in fertility rates and the demographic transition process that Brazil is undergoing^(23)^. In addition, the possibility that the increase in occurrence is related to improved diagnosis due to the increased availability of obstetric ultrasound should be considered. Since its introduction in medicine, this technology has evolved significantly, generating increasingly accurate and detailed images, allowing for the monitoring of fetal development and the detection of malformations, with the possibility of screening for Down syndrome and major structural abnormalities as early as the first trimester, and other serious fetal anomalies from the second trimester onwards^(24)^.

Regarding the category of fetuses and newborns affected by maternal factors and complications of pregnancy, labor, and delivery, an international study found that maternal conditions were responsible for 24.2% of fetal deaths, ranking second among the main causes of these deaths. Among these conditions, the main one included was hypertensive disease of pregnancy (82.5%), and the authors related the predominance of this cause to the ethnic composition of the population, which is predominantly Afro-Caribbean, in addition to low socioeconomic status. The primary cause associated with stillbirths was obstetric complications, more specifically, asphyxia associated with labor (57%) and premature placental abruption (26%), which in this study was associated with hypertensive disorders in 47% of patients^(20)^. Another study conducted in India also highlighted that the maternal condition most associated with stillbirths was hypertensive disease of pregnancy, ranging from 6.4% to 27.6%.

Most of the maternal conditions associated with the occurrence of these deaths, as well as complications of pregnancy, labor, and delivery, are modifiable through early diagnosis and appropriate treatment through quality prenatal and delivery care^(25)^. Given this, the relevance of this category is highlighted, since approximately 40% of the deaths analyzed in this investigation were associated with it, suggesting a failure in the monitoring, diagnosis, and early treatment of past and current maternal health problems, fetal complications of the placenta and adnexa, complications of labor, and complications related to maternal habits. The increase in the trend of this basic cause in the North and Northeast regions can be explained, in addition to population characteristics, by the social inequalities to which they are subjected, demonstrated by the higher proportion of no consultations and the lower proportion of seven or more prenatal consultations^(18).^

Regarding congenital malformations of the circulatory system, an increase was observed among intermediate and late deaths. National and international studies have shown that, among deaths due to congenital malformations, those of the circulatory system were the most frequent (35.3% and 32.0%), respectively^(26,27)^. Even though most congenital anomalies result in fetal death, many of them are potentially treatable if diagnosed early, which can improve outcomes in anomalies known to be non-lethal^(28)^.

As for other congenital malformations and deformities of the musculoskeletal system, with an increase in the period for early and late fetal deaths, the study described that among 46.705 live births, 485 had congenital malformations, and of these, 42.1% were related to the musculoskeletal system, which was the most prevalent congenital malformation among the live births analyzed in the study. The authors point out that this may be because these conditions are visible and easily detectable on physical examination, resulting in early diagnosis in the immediate postnatal period. They also point out that, because they have a significant influence on the infant mortality rate, congenital malformations represent a public health problem, thus requiring adequate and qualified attention in order to prevent and reduce morbidity and mortality^(26)^.

Finally, regarding congenital syphilis, as it is a preventable disease that is easily identifiable and treatable in the public health service, the identified occurrence is considered high (1.4%), which is especially concerning due to the statistically significant increase in late fetal deaths. Brazilian data show that the detection rates of pregnant women with syphilis have continued to grow between 2011 and 2022, but with less intensity since 2018: between 2011 and 2017, the average growth was 17.6%, followed by stability in subsequent years and an increase of 16.7% in 2021^(29)^.

A national study found that of the 558 deaths recorded for syphilis, toxoplasmosis, rubella, cytomegalovirus, and herpes simplex virus, 98.7% were due to congenital syphilis. It is worth noting that syphilis is a disease whose diagnosis and treatment are low-cost and easily accessible, which, when offered in a timely manner, can lead to complete remission of the disease in the mother, preventing vertical transmission and avoiding the possibility of fetal deaths due to this condition^(17)^. Given this, syphilis is an indicator of the quality of prenatal care, and deaths related to this disease are considered unacceptable even in the most precarious health systems, suggesting deficiencies in the quality of prenatal care offered to pregnant women, problems with coverage in the provision of diagnosis, or even poor or non-existent adherence to treatment for syphilis^(30)^.

A weakness of the present study is the fact that it deals with secondary data obtained exclusively from the SIM and, as such, it was not possible to directly control its quality, with possible bias regarding the underlying cause of death due to underreporting or coding errors in the death certificate, as well as incomplete diagnosis records being particularly relevant. It was also not possible to assess the causality related to deaths, due to the observational design adopted, nor to evaluate clinical, maternal, and fetal variables not recorded in the death certificate and the impact of public health interventions that occurred during the study period.

On the other hand, the use of secondary data allowed us to address data from Brazil over a decade, a situation that is especially relevant given the country’s continental dimensions, as well as the possibility of stratifying fetal mortality according to gestational age and underlying cause of death, aspects not addressed in previous national studies.

The vast majority of fetal deaths occurred due to potentially preventable causes, mainly through perinatal monitoring, including early diagnosis and treatment of maternal and fetal diseases. Thus, it is hoped that this study will support health managers and professionals who care for mothers and children, particularly in the areas of nursing, obstetrics, neonatology, and family health, as their actions can directly impact this reality through the development of health education initiatives, systematic prenatal monitoring, early identification of maternal-fetal risk signs, implementation of protocols for the management of complications during labor, and strengthening of epidemiological surveillance at different points in the Health Care Network.

For managers, it is recommended to prioritize the quality of prenatal care, ensuring an adequate number of consultations, routine ultrasounds, and screening for maternal diseases such as hypertension and diabetes. It is essential to strengthen the perinatal care network, ensuring early referral of pregnant women to the appropriate maternity hospital and the availability of up-to-date clinical protocols for the management of complications during labor and delivery. The expansion of continuing education initiatives for health teams, the use of sensitive epidemiological surveillance systems, such as timely investigation of fetal deaths and monitoring of quality indicators for prenatal and delivery care, can be incorporated as management goals. Such measures can reduce regional inequalities, improve the coordination of the maternal and child care network, and have a significant impact on reducing fetal mortality.

## CONCLUSION

Most fetal deaths between 2011 and 2020 are included in Chapter 16 of ICD-10 (some conditions originating in the perinatal period), with the following groups standing out: fetus and newborn affected by maternal factors and complications of pregnancy, labor, and delivery; intrauterine hypoxia and asphyxia at birth, and other conditions originating in the perinatal period. Hypoxia and asphyxia are the only category showing a downward trend for all types of death (early, intermediate, or late). However, there was an upward trend in the underlying causes: Chromosomal anomalies not classified elsewhere and Fetus and newborn affected by maternal factors and complications of pregnancy, labor, and delivery, also for early, intermediate, or late deaths.

It should be noted that the vast majority of causes of death identified in this study are characterized as preventable through adequate prenatal, delivery, and newborn care, which highlights the need to improve perinatal care in order to reduce and/or prevent their occurrence. These results can inform public health policies aimed at preventing fetal deaths, with a special focus on conditions that still show an upward trend.

## Data Availability

The entire dataset supporting the results of this study is available upon request to the corresponding author.
