# Supplementary material for: Temporal behavior of proportional mortality of fetal deaths according to underlying cause, 2011–2020
Source: Rev Esc Enferm USP. 2026 Feb 16;60:e20250288. doi: 10.1590/1980-220X-REEUSP-2025-0288en (PMC12919764; doi:10.1590/1980-220X-REEUSP-2025-0288en)
Supplement: Translation

# Translation: Comportamento temporal da mortalidade proporcional dos óbitos fetais segundo causa básica, 2011–2020

**DOI:** 10.1590/1980-220X-REEUSP-2025-0288pt

**Authors:** Júlia Rodrigues Soares de Barros, Ana Beatriz Henrique Parenti, Ana Paula Pinho Carvalheira, Anna Paula Ferrari, Marli Teresinha Cassamassimo Duarte, Cristina Maria Garcia de Lima Parada

**Affiliations:** 1 Universidade Estadual Paulista “Júlio de Mesquita Filho”, Faculdade de Medicina de Botucatu, Departamento de Enfermagem, Botucatu, SP, Brasil.

**Keywords:** Morte Fetal, Epidemiologia, Fatores de Risco, Sistemas de Informação em Saúde

## Abstract

**Objetivo:**

Investigar o comportamento temporal da mortalidade proporcional dos óbitos fetais precoces, intermediários e tardios entre 2011-2020, segundo causa básica.

**Métodos:**

Estudo de base populacional realizado com dados secundários de óbitos fetais registrados no Sistema de Informações de Mortalidade referentes ao período entre 2011–2020.

**Resultados:**

As maiores médias anuais da mortalidade proporcional segundo causa básica dos óbitos fetais precoces, intermediários ou tardios foram, respectivamente: feto e recém-nascido afetado por fatores maternos e complicações da gravidez, trabalho de parto e parto (41,3%, 44,4% e 44,0%); hipóxia intrauterina e asfixia ao nascer (22,3%, 21,6% e 22,8%) e outras afecções originadas no período perinatal (28,0%, 23,4% e 24,4%). Hipóxia e asfixia apresentaram tendência de diminuição para todos os tipos de óbito. O grupo Feto e recém-nascido afetado por fatores maternos e por complicações da gravidez, trabalho de parto e parto, apresentou tendência de aumento.

**Conclusão:**

As principais causas básicas de óbitos são caracterizadas como evitáveis pela adequada atenção ao pré-natal, parto e recém-nascido, o que evidencia a necessidade de qualificar o cuidado perinatal, visando diminuir e/ou evitar sua ocorrência.

## INTRODUÇÃO

A Organização Mundial da Saúde (OMS) e a Classificação Estatística Internacional de Doenças e Problemas Relacionados à Saúde – 11ª revisão (CID-11) definem óbito fetal como a morte de um feto antes da sua expulsão ou extração completa do corpo de uma mulher, independentemente da duração da gestação^(1)^. Constitui significativo problema de saúde pública mundial e importante indicador da saúde materno-infantil^(2)^, por refletir a qualidade da assistência e as condições de acesso aos cuidados de saúde disponibilizados às gestantes desde o pré-natal até o momento do desfecho do parto^(3,4)^.

Pode-se classificar o óbito fetal como precoce (inferior a 20 semanas de gestação), intermediário (entre 20 e 28 semanas) e tardio (após 28 semanas), ou então como anteparto ou intraparto, de acordo com o período de ocorrência^(5)^, sendo que esse desfecho negativo é mais frequente em países de média e baixa renda^(6)^. Em países de alta renda, como os Estados Unidos da América, a melhoria do atendimento a gestantes no último século resultou em redução importante nos óbitos fetais e queda proporcionalmente maior nos óbitos neonatais, de forma que a morte fetal continua a ser um problema significativo e pouco estudado, responsável por quase 50% de todas as mortes perinatais no país^(7)^. No Brasil, a taxa de mortalidade fetal global no período entre 1996 e 2021 foi 11,4/1000 nascidos vivos, com todas as regiões apresentando tendência de queda, com algumas flutuações pontuais, sendo a queda mais acentuada registrada na região Sudeste, com redução de 48%^(8)^.

A morte fetal pode ser influenciada pela ocorrência simultânea de diversos fatores e esse caráter multifatorial pode dificultar a detecção da causa específica^(9)^. Dentre os fatores associados a resultados perinatais adversos, inclusive o óbito fetal, estão: idade materna superior a 35 anos; nuliparidade; tabagismo; perdas fetais anteriores; doenças maternas prévias, como hipertensão arterial, diabetes *mellitus* e obesidade; infecções maternas na gestação; complicações placentárias, umbilicais, amnióticas ou uterinas; anomalias congênitas; asfixia e traumas ao nascimento; restrição do crescimento fetal e a ausência ou má-qualidade da assistência pré-natal. Além disso, alguns fatores sociais, como baixa escolaridade, cor da pele materna preta ou parda e condições socioeconômicas precárias também se associam aos óbitos fetais^(10)^.

Estudo nacional sobre óbitos fetais ocorridos entre 1996 e 2021^(8)^ os apresentou a partir da taxa de mortalidade global, não considerando a classificação segundo idade gestacional de ocorrência, lacuna que se pretende abordar no presente estudo, que objetiva investigar o comportamento temporal da mortalidade proporcional dos óbitos fetais precoces, intermediários e tardios entre 2011–2020, segundo sua causa básica e macrorregião de ocorrência, com vistas a subsidiar ações para melhorar a saúde materno-infantil e a adoção de medidas preventivas, que evitem sua ocorrência.

## MÉTODO

### Desenho do Estudo

Trata-se estudo observacional, retrospectivo, de base populacional, realizado a partir de dados secundários do Sistema de Informação sobre Mortalidade (SIM) sobre óbitos fetais. Em sua construção foi utilizada a Declaração STROBE^(11)^.

### População e Fonte de Dados

Foram incluídos no estudo todos os casos de óbito fetal ocorridos no Brasil e registrados no SIM no período de 2011 a 2020. O SIM constitui sistema de vigilância epidemiológica nacional, que tem por objetivo captar dados sobre os óbitos ocorridos no país, sendo seu documento básico a Declaração de Óbito (DO), cujo uso é obrigatório, com o objetivo de que as estatísticas de mortalidade produzidas possam ser válidas e comparáveis. O Sistema apresenta completude classificada como excelente (menos de 5% de incompletude) e, apesar das disparidades na qualidade de suas diferentes dimensões, estudos indicam melhorias ao longo do tempo nos registros de dados de mortalidade da população brasileira^(12)^.

### Variáveis do Estudo

Foram utilizadas variáveis relacionadas às características maternas e fetais, dados da gravidez e parto, e relativos ao óbito: Maternas: idade (anos completos); escolaridade (até 3, 4–7, 8–11, 12 ou mais); atividade remunerada (sim, não); número de filhos vivos (0–3, ≥4); filhos mortos (sim, não); região de residência (Norte, Nordeste, Centro-oeste, Sudeste, Sul). Fetais: sexo (masculino, feminino); peso ao nascer (<1500, 1500–2499, ≥2500); raça/cor de pele (Branca, Parda, Preta, Amarela, Indígena). Dados da gravidez e parto: tipo de gravidez (única, múltipla); tipo de parto (vaginal, cesárea); idade gestacional (semanas completas). Dados relativos ao óbito: trimestre de ocorrência (primeiro, segundo e terceiro); momento do óbito (antes do parto, depois do parto); macrorregião de ocorrência (norte, nordeste, centro-oeste, sudeste, sul); assistência médica ao óbito (sim, não). Causa básica por Grupo, segundo capítulo da CID-10^(13)^: Capítulos: I - doenças infecciosas e parasitárias; II - neoplasias/tumores; III - doenças do sangue, dos órgãos hematopoiéticos e alguns transtornos imunitários; IV - doenças endócrinas, nutricionais e metabólicas; V - transtornos mentais e comportamentais; VI - doenças do sistema nervoso; VII - doenças do olho e anexos; VIII - doenças do ouvido e da apófise mastoide; IX - doenças do aparelho circulatório; X - doenças do aparelho respiratório; XI - doenças do aparelho digestivo; XII - doenças da pele e do tecido subcutâneo; XIII - doenças do sistema osteomuscular e do tecido conjuntivo; XIV - doenças do aparelho geniturinário; XV - gravidez, parto e puerpério; XVI - algumas afecções originadas no período perinatal; XVII - malformações congênitas, deformidades e anomalias cromossômicas; XVIII - sintomas, sinais e achados anormais de exames clínicos e de laboratório, não classificados em outra parte; XIX - lesões, envenenamentos e algumas outras consequências de causas externas; XX - causas externas de morbidade e de mortalidade; XXI - fatores que exercem influência sobre o estado de saúde e o contato com serviços de saúde; XXII - códigos para propósitos especiais.

### Métodos Estatísticos

Inicialmente, foi realizada análise descritiva das variáveis relativas às características maternas e fetais, da gravidez, parto e óbito. Para investigação da existência de tendência de aumento ou diminuição da mortalidade proporcional dos óbitos fetais por causa básica e macrorregião de ocorrência, nas populações de óbito fetal precoce, intermediário e tardio, foram ajustados modelos de regressão linear com resposta normal para cada série temporal de mortalidade proporcional, considerando o tempo como variável independente. Além disso, a tendência de cada série foi testada por meio do teste de Cox-Stuart. Análises foram feitas com o software SPSS 21.

### Aspectos Éticos

Foi assegurada a preservação dos aspectos éticos, de acordo com a Resolução do Conselho Nacional de Saúde n^o^ 510, de 7 de abril de 2016, parágrafo único, que dispensa o registro e avaliação pelo sistema Comitê de Ética em Pesquisa/Comissão Nacional de Ética em Pesquisa (CEP/CONEP) as pesquisas que utilizem informações de acesso público^(14)^.

## RESULTADOS

Na década 2011-2020 ocorreram 164.504 óbitos fetais registrados no SIM. O Capítulo 16 da CID-10 (algumas afecções originadas no período perinatal) incluiu a maior parte deles, seguido pelo Capítulo 17 (malformações congênitas, deformidades e anomalias cromossômicas) e Capítulo 1 (Algumas doenças infecciosas e parasitárias), conforme consta da Tabela 1.

**Tabela 1:** Descrição dos óbitos por capítulo da CID-10 – Brasil, 2011–2020

Capítulo	Categoria	n	%
**1 doenças infecciosas e parasitárias**			
Sífilis congênita		2340	1,4
**16 algumas afecções originadas no período perinatal**			
Feto e recém-nascido afetado por fatores maternos e por complicações da gravidez, trabalho de parto e parto		72436	44,0
Outras afecções originadas no período perinatal		40138	24,4
Hipóxia intrauterina e asfixia ao nascer		36891	22,4
Restrição no crescimento fetal, desnutrição fetal e transtornos relacionados à gestação curta e baixo peso ao nascer		1844	1,1
Doença hemolítica do feto e do recém-nascido		266	0,2
Outras infecções específicas do período perinatal		277	0,2
Doenças infecciosas e parasitárias congênitas		175	0,1
Trauma durante o nascimento		98	0,06
Outros transtornos respiratórios originados no período perinatal		48	0,03
**17 malformações congênitas, deformidades e anomalias cromossômicas**		3997	2,4
Outras malformações congênitas			
Outras malformações congênitas do sistema nervoso		2409	1,5
Anomalias cromossômicas, não classificadas em outra parte		1049	0,7
Malformações congênitas do aparelho circulatório		1247	0,7
Outras malformações e deformidades congênitas do aparelho osteomuscular		662	0,4
Outras malformações do aparelho geniturinário		377	0,2
Outras malformações congênitas do aparelho digestivo		129	0,08
Espinha bífida		74	0,04
Fenda labial e fenda palatina		31	0,02
Ausência, atresia e estenose do intestino delgado		16	0,01

Em relação às médias anuais da mortalidade proporcional, segundo causa básica, destacaram-se as seguintes categorias, considerando-se a ocorrência de óbito fetal precoce, intermediário ou tardio, respectivamente: feto e recém-nascido afetado por fatores maternos e complicações da gravidez, trabalho de parto e parto (41,3%, 44,4% e 44,0%); hipóxia intrauterina e asfixia ao nascer (22,3%, 21,6% e 22,8%) e outras afecções originadas no período perinatal (28,0%, 23,4% e 24,4%), todas do Capítulo 16 da CID 10 (dados não apresentados em tabela).

Hipóxia intrauterina e asfixia ao nascer tiveram tendência de diminuição para todos os tipos de óbito: precoce, intermediário e tardio. Também houve tendência à diminuição para outras infecções específicas do período perinatal em caso de óbitos fetais intermediários e outras malformações do sistema nervoso, entre óbitos fetais tardios (Tabela 2).

**Tabela 2:** Investigação da tendência da mortalidade fetal proporcional segundo causa básica de morte – Brasil, 2011–2020.

Categoria	Precoce			Intermediária			Tardia		
	β(IC95%)	*p*	Tendência	β(IC95%)	*p*	Tendência	β(IC95%)	*p*	Tendência
Anomalias cromossômicas, não classificadas em outra parte	0,05 (0,01–0,09)	*0,019*	Aumento	0,05 (0,03–0,08)	*0,001*	Aumento	0,06 (0,04–0,07)	*<0,001*	Aumento
Ausência, atresia e estenose do intestino delgado	0,00 (–0,01–0,01)	*0,873*	Estacionário	0,00 (0,00–0,00)	*0,244*	Estacionário	0,00 (0,00–0,00)	*0,700*	Estacionário
Doença hemolítica do feto e do recém-nascido	0,00 (–0,02–0,02)	*0,752*	Estacionário	0,01 (–0,01–0,02)	*0,223*	Estacionário	0,00 (–0,01–0,00)	*0,194*	Estacionário
Doenças infecciosas e parasitárias congênitas	–0,02 (–0,04–0,00)	*0,074*	Estacionário	0,00 (–0,01–0,01)	*0,778*	Estacionário	0,00 (0,00–0,01)	*0,168*	Estacionário
Espinha bífida	0,01 (–0,01–0,02)	*0,388*	Estacionário	0,01 (–0,01–0,02)	*0,287*	Estacionário	0,00 (–0,01–0,00)	*0,274*	Estacionário
Fenda labial e fenda palatina	0,00 (–0,01–0,00)	*0,122*	Estacionário	0,00 (0,00–0,00)	*0,997*	Estacionário	0,00 (0,00–0,00)	*0,491*	Estacionário
Feto e recém-nascido afetado por fatores maternos e por complicações da gravidez, trabalho de parto e parto	0,74 (0,26–1,21)	*0,007*	Aumento	0,53 (0,27–0,78)	*0,001*	Aumento	0,53 (0,28–0,79)	*0,001*	Aumento
Hipóxia intrauterina e asfixia ao nascer	–0,44 (–0,81– –0,07)	*0,024*	Diminuição	–0,48 (–0,67– –0,28)	*<0,001*	Diminuição	–0,55 (–0,84– –0,26)	*0,002*	Diminuição
Malformações congênitas do aparelho circulatório	0,01 (–0,04–0,06)	*0,615*	Estacionário	0,05 (0,02–0,07)	*0,004*	Aumento	0,04 (0,02–0,07)	*0,002*	Aumento
Outras afecções originadas no período perinatal	–0,31 (–0,92–0,30)	*0,273*	Estacionário	–0,19 (–0,47–0,09)	*0,151*	Estacionário	–0,19 (–0,67–0,29)	*0,394*	Estacionário
Outras infecções específicas do período perinatal	0,02 (–0,02–0,05)	*0,340*	Estacionário	–0,02 (–0,03– –0,01)	*0,002*	Diminuição	–0,01 (–0,02–0,00)	*0,077*	Estacionário
Outras malformações congênitas	–0,03 (–0,11–0,05)	*0,360*	Estacionário	0,04 (–0,06–0,13)	*0,401*	Estacionário	0,00 (–0,04–0,03)	*0,889*	Estacionário
Outras malformações congênitas do aparelho digestivo	0,00 (–0,01–0,01)	*0,737*	Estacionário	0,00 (–0,01–0,01)	*0,550*	Estacionário	0,00 (–0,01–0,00)	*0,035*	Irrelevante
Outras malformações congênitas do sistema nervoso	0,05 (–0,01–0,11)	*0,107*	Estacionário	0,02 (–0,06–0,09)	*0,613*	Estacionário	–0,05 (–0,08– –0,03)	*0,002*	Diminuição
Outras malformações do aparelho Geniturinário	0,02 (–0,02–0,07)	*0,278*	Estacionário	0,00 (–0,03–0,03)	*0,913*	Estacionário	0,01 (0,00–0,03)	*0,089*	Estacionário
Outras malformações e deformidades congênitas do aparelho osteomuscular	0,03 (0,00–0,06)	*0,038*	Aumento	0,01 (–0,01–0,02)	*0,535*	Estacionário	0,02 (0,00–0,03)	*0,027*	Aumento
Outros transtornos respiratórios originados no período perinatal	0,00 (–0,01–0,01)	*0,742*	Estacionário	0,00 (–0,01–0,01)	*0,633*	Estacionário	0,00 (–0,01–0,00)	*0,794*	Estacionário
Retardo no crescimento fetal, desnutrição fetal e transtornos relacionados à gestação curta e baixo peso ao nascer	–0,15 (–0,36–0,06)	*0,127*	Estacionário	–0,04 (–0,18–0,09)	*0,479*	Estacionário	0,03 (0,00–0,06)	*0,085*	Estacionário
Sífilis congênita	0,03 (–0,03–0,08)	*0,345*	Estacionário	0,04 (–0,06–0,15)	*0,400*	Estacionário	0,13 (0,06–0,19)	*0,003*	Aumento
Trauma durante o nascimento	0,00 (–0,01–0,02)	*0,721*	Estacionário	–0,01 (–0,02–0,00)	*0,119*	Estacionário	0,00 (–0,01–0,01)	*0,295*	Estacionário

As categorias que apresentaram aumento estatisticamente significativo na tendência para todos os tipos de óbito foram: anomalias cromossômicas não classificadas em outra parte e feto e recém-nascido afetado por fatores maternos e complicações da gravidez, trabalho de parto e parto. Houve tendência de aumento no caso dos óbitos fetais precoces para outras malformações e deformidades congênitas osteomuscular. Para os óbitos intermediários e tardios houve tendência de aumento para malformações congênitas do aparelho circulatório e apenas entre os óbitos tardios ainda houve tendência de aumento para outras malformações e deformidades congênitas do aparelho osteomuscular e sífilis congênita (Tabela 2).

Entre os óbitos fetais precoces, as séries temporais da mortalidade proporcional segundo as categorias: anomalias cromos-sômicas não classificadas em outra parte; feto e recém-nascido afetado por fatores maternos e por complicações da gravidez, trabalho de parto e parto e outras malformações e deformidades congênitas osteomuscular apresentaram tendência de aumento anual, enquanto que a série da mortalidade proporcional segundo a categoria hipóxia intrauterina e asfixia ao nascer apresentou tendência de diminuição anual (Figura 1).

**Figura 1:** Análise da tendência anual dos óbitos fetais precoces segundo macrorregião de ocorrência e categoria da CID-10.

No que diz respeito à mortalidade proporcional dos óbitos fetais precoces, segundo macrorregiões, observou-se aumento nas categorias: anomalias cromossômicas na região Sul (β = 0,23, IC95% = 0,04–0,41, p = 0,021); feto e recém-nascido afetado por fatores maternos e por complicações da gravidez, trabalho de parto e parto na região norte (β = 2,10, IC95% = 0,82–3,38, p = 0,005) e tendência de diminuição na categoria hipóxia intrauterina e asfixia ao nascer nas regiões centro-oeste e sul, respectivamente (β = -1,05, IC95%= –1,84 – –0,25, p = 0,017) e (β = –1,00, IC95%= –1,89 – –0,11, p = 0,032). Não houve resultados significativos na categoria outras malformações e deformidades congênitas do aparelho osteomuscular por macrorregião (Figura 1).

Entre os óbitos fetais intermediários, as séries temporais da mortalidade proporcional segundo as categorias: anomalias cromossômicas não classificadas em outra parte; feto e recém-nascido afetado por fatores maternos e por complicações da gravidez, trabalho de parto e parto e malformações congênitas do aparelho circulatório sofreram aumento, enquanto que as categorias hipóxia intrauterina e asfixia ao nascer e outras infecções específicas do período perinatal diminuiram no mesmo período (Figura 2).

**Figura 2:** Análise da tendência anual dos óbitos fetais intermediários segundo macrorregião de ocorrência e categoria da CID-10.

Sobre a mortalidade proporcional dos óbitos fetais intermediários, segundo macrorregiões, observou-se aumento nas categorias: anomalias cromossômicas nas regiões sul e centro-oeste (β = 0,20, IC95% = 0,08–0,31, p = 0,004) e (β = 0,11, IC95% = 0,004–0,18, p = 0,006), respectivamente; feto e recém-nascido afetado por fatores maternos e por complicações da gravidez, trabalho de parto e parto nas regiões norte (β = 2,88, IC95% = 2,34–3,42, p < 0,001) e nordeste (β = 0,50, IC95% = 0,21–0,79, p = 0,004) e sífilis congênita na região centro-oeste (β = 0,17, IC95% = 0,08–0,27, p = 0,002). Houve tendência de diminuição na categoria hipóxia intrauterina e asfixia ao nascer nas regiões nordeste, centro-oeste e sul (β = –0,77, IC95% = –1,21– –0,33, p = 0,004), (β = –0,95, IC95% = –1,52 – –0,38, p=0,005) e (β = –0,67, IC95% = –1,08 – –0,25, p = 0,006), respectivamente. Não houve resultados significativos na categoria outras infecções específicas do período perinatal e malformações congênitas do aparelho circulatório, quando considerada a macrorregião de ocorrência (Figura 2).

Entre os óbitos fetais tardios, as séries temporais da mortalidade proporcional segundo as categorias: anomalias cromos-sômicas não classificadas em outra parte; feto e recém-nascido afetados por fatores maternos e por complicações da gravidez, trabalho de parto e parto; malformações congênitas do aparelho circulatório; outras malformações e deformidades congênitas do aparelho osteomuscular e sífilis congênita da CID-10 apresentaram tendência de aumento anual, enquanto que a série da mortalidade proporcional segundo a categoria: hipóxia intrauterina e asfixia ao nascer e outras malformações congênitas do sistema nervoso apresentou tendência de diminuição anual (Figura 3).

**Figura 3:** Análise da tendência anual dos óbitos fetais tardios segundo macrorregião de ocorrência e categoria da CID-10.

Sobre a mortalidade proporcional dos óbitos fetais tardios, segundo macrorregiões, observou-se aumento nas categorias anomalias cromossômicas nas regiões nordeste (β = 0,02, IC95% = 0,00–0,04, p = 0,036, centro-oeste (β = 0,10, IC95% = 0,04– 0,17, p = 0,0050, sudeste (β = 0,06, IC95% = 0,02–0,11, p = 0,010) e sul (β = 0,17, IC95% = 0,10–0,23, p < 0,001); feto e recém-nascido afetado por fatores maternos e por complicações da gravidez, trabalho de parto e parto nas regiões norte (β = 2,35, IC95% = 1,83–2,87, p < 0,001) e nordeste (β = 0,42, IC95% = 0,00–0,84, p = 0,005); malformação do aparelho circulatório nas regiões norte (β = 0,06, IC95% = 0,02-0,10, p = 0,005), nordeste (β = 0,06, IC95% = 0,01–0,11, p = 0,035) e sudeste (β = 0,03, IC95% = 0,00–0,06, p = 0,027); malformação sistema nervoso nas regiões nordeste (β = –0,07, IC95% = –0,11 – –0,03, p = 0,002) e sul (β = –0,14, IC95% = –0,22 – –0,06, p=0,004); malformação osteomuscular nas regiões norte (β = 0,06, IC 95% = 0,03–0,09, p = 0,002) e sul (β = 0,06, IC95% = 0,01–0,10, p = 0,014) e sífilis nas regiões centro-oeste (β = 0,25, IC95% = 0,18–0,32, p < 0,001) e sudeste (β = 0,20, IC95% = 0,11–0,29, p = 0,001). Houve tendência de diminuição na categoria hipóxia intrauterina e asfixia ao nascer nas regiões nordeste (β =.–0,82, IC95% = –1,18 – –0,46, p = 0,001), centro-oeste (β = –0,79, IC95%= –1,10 – –0,47, p < 0,001) e sul (β = –0,70, IC95% = –0,97 – –0,43, p < 0,001) (Figura 3).

## DISCUSSÃO

Este estudo permitiu conhecer o comportamento temporal da mortalidade proporcional dos óbitos fetais segundo causa básica no período de 2011 a 2020, sendo que o capítulo 16 da CID-10 (algumas afecções originadas no período perinatal) incluiu a maior parte dos óbitos fetais ocorridos: 92,5%, seguido do capítulo 17 (malformações congênitas, deformidades e anomalias cromossômicas): 6,1% e capítulo 1 (doenças infecciosas e parasitárias): 1,4%.

Pelo exposto, foi possível identificar tendência de aumento na causa básica feto e recém-nascido afetado por fatores maternos e por complicações da gravidez, trabalho de parto e parto nas regiões Norte e Nordeste e, quanto às demais causas, não foi possível identificar um padrão de aumento ou diminuição na tendência da mortalidade fetal proporcional, segundo causa básica de óbito, de acordo com as diferentes regiões do país.

Estudo que analisou os óbitos fetais ocorridos na região centro-oeste entre 2008–2018 mostrou que 93% dos óbitos incluídos no estudo estavam associados ao capítulo 16 da CID-10 (algumas afecções originadas no período perinatal), achado semelhante ao do presente estudo, onde 92,5% dos óbitos também se associaram ao capítulo 16. Destaca-se que a grande maioria das afecções originadas no período perinatal são suscetíveis à prevenção, o que sugere a necessidade de melhoria na qualidade da assistência prestada, tanto no pré-natal, quanto no parto^(15,16)^.

A asfixia perinatal, que inclui a hipóxia intrauterina e a asfixia ao nascer, apresentou tendência de diminuição para todos os tipos de óbitos. Estudo que analisou a epidemiologia da mortalidade perinatal, segundo a classificação de Wigglesworth, destacou que, dos 58,9% de óbitos fetais, apenas 0,8% tiveram como causa a asfixia^(17)^. A relevância desta categoria, no presente estudo, decorre da elevada frequência identificada no período, visto que aproximadamente 20% dos óbitos tiveram essa causa básica. Diante disso, o resultado da tendência de diminuição na ocorrência dos óbitos fetais precoces, intermediários e tardios relacionados a essa causa sinaliza que a qualidade da assistência prestada, tanto à gestante quanto ao feto, pode ter melhorado ao longo do período estudado. Estudo que teve por objetivo analisar indicadores relacionados ao pré-natal, utilizando as informações das edições da Pesquisa Nacional de Saúde de 2013 e 2019, encontrou aumento significativo no número de consultas pré-natais e de exames clínico-obstétricos, na proporção de mulheres que receberam orientações sobre o serviço de referência para o parto e na realização de testes para sífilis e HIV. Os autores concluíram que houve avanços importantes na cobertura e na qualidade do atendimento às gestantes no Brasil, embora persistam desafios como o início tardio do pré-natal e ainda insuficiente orientação sobre os serviços de referência para o parto^(18)^.

A diminuição na ocorrência de óbitos fetais por malformações do sistema nervoso pode decorrer da introdução da suplementação de ácido fólico no período periconcepcional e durante o primeiro trimestre gestacional, que resultou na diminuição de aproximadamente 50% dos defeitos do tubo neural, bem como o diagnóstico precoce de malformações a partir de exames de imagem realizados durante a gestação e novas ténicas neuro-cirúrgicas intrauterinas, melhorando o resultado perinatal^(19)^.

No que se refere às anomalias cromossômicas não classificadas em outra parte, estudo realizado na Guiana Francesa destacou que 6% dos óbitos ocorridos foram devidos a anomalias fetais, onde quase 50% desses óbitos foram decorrentes de anomalias cromossômicas^(20)^ e estudo realizado no nordeste brasileiro, evidenciou que, apesar de 2,9% dos óbitos fetais terem ocorrido por malformações congênitas, houve aumento estatisticamente significativo desses casos^(21)^. Estudo ecológico de base populacional realizado no Brasil comparou as malformações ocorridas entre mulheres com idades entre 35–40 anos e acima de 41 anos e, ao contrário do que se esperava, quando foram analisadas as malformações por subgrupo etário, evidenciou-se tendência de aumento apenas entre as mais jovens, destacando os autores, que esse achado pode decorrer do pequeno tamanho do subgrupo com mais idade^(22)^.

Como há evidências de associação entre malformação e idade materna avançada, um dos fatores que pode estar relacionado ao citado aumento é a opção das mulheres por adiar a gestação, decorrente do processo de urbanização e do seu interesse pela escolarização, fatores relacionados à queda na taxa de fecundidade e ao processo de transição demográfica pelo qual passa o Brasil^(23)^. Além disso, deve-se considerar a possibilidade de o aumento na ocorrência estar relacionado à melhora diagnóstica pelo aumento na disponibilidade de uso da ultrassonografia obstétrica. Desde sua introdução na medicina, essa tecnologia tem evoluído significativamente, gerando imagens cada vez mais precisas e detalhadas, permitindo o acompanhamento do desenvolvimento fetal e a detecção de malformações, com possibilidade de rastrear a síndrome de Down e grandes anormalidades estruturais, já no primeiro trimestre e, outras anomalias fetais graves, à partir do segundo trimestre^(24)^.

Quanto à categoria feto e recém-nascido afetados por fatores maternos e por complicações da gravidez, do trabalho de parto e do parto, estudo internacional apontou que as condições maternas foram responsáveis por 24,2% das mortes fetais, ficando em segundo lugar nas principais causas desses óbitos. Dentre estas condições, a principal incluída foi a doença hipertensiva da gravidez (82,5%) e os autores relacionaram a predominância dessa causa com a composição étnica da população, majorita-riamente afro-caribenha, além do baixo nível socioeconômico. A primeira causa associada à ocorrência de natimortos foram as complicações obstétricas, mais especificamente, a asfixia associada ao trabalho de parto (57%) e o descolamento prematuro de placenta (26%), que nesse estudo associou-se aos distúrbios hipertensivos em 47% das pacientes^(20)^. Outro estudo realizado na Índia também destacou que a condição materna mais associada à ocorrência de natimortos foi a doença hipertensiva da gravidez, oscilando de 6,4% a 27,6%.

A maior parte das condições maternas associadas à ocorrência destes óbitos, bem como as complicações da gravidez, trabalho de parto e parto, são modificáveis a partir do diagnóstico precoce e tratamento adequado por meio do cuidado pré-natal e ao parto de qualidade^(25)^. Diante disso, destaca-se a relevância desta categoria, visto que, aproximadamente, 40% dos óbitos analisados na presente investigação associaram-se a ela, o que sugere a ocorrência de falha em relação ao acompanhamento, diagnóstico e tratamento precoce de problemas de saúde maternos pregressos e atuais, complicações fetais da placenta e anexos, complicações do trabalho de parto e complicações relacionadas a hábitos maternos. O aumento da tendencia dessa causa básica nas regiões Norte e Nordeste pode ser justificado, para além das características populacionais, pelas iniquidades sociais a que estão submetidas, demonstradas pela maior proporção de nenhuma consulta e menor proporção de realização de sete ou mais consultas de pré-natal^(18).^

Sobre as malformações congênitas do aparelho circulatório, observou-se aumento entre óbitos intermediários e tardios. Estudos nacional e internacional demonstraram que, dentre os óbitos por malformações congênitas, as do aparelho circulatório eram as mais frequentes (35,3% e 32,0%), respectivamente^(26,27)^. Mesmo que grande parte dos desfechos das anomalias congênitas seja o óbito fetal, grande parte destas são potencialmente tratáveis se realizado diagnóstico precoce, podendo melhorar os resultados nas anomalias conhecidas como não letais^(28)^.

Quanto às outras malformações e deformidades congênitas do aparelho osteomuscular, com aumento no período para os óbitos fetais precoces e tardios, estudo descreveu que entre 46.705 nascidos vivos, 485 possuíam malformação congênita e, destes, 42,1% eram relacionadas ao aparelho osteomuscular, sendo esta a malformação congênita mais predominante entre os nascidos vivos analisados no estudo. Os autores destacam que isso pode se dar por serem condições visíveis e detectáveis facilmente ao exame físico, o que resulta em diagnóstico precoce no período pós-natal imediato. Também destacam que, por influenciarem de forma relevante a taxa de mortalidade infantil, as malformações congênitas representam problema de saúde pública, precisando, assim, de atenção adequada e qualificada, a fim de prevenir e reduzir a morbimortalidade^(26)^.

Por fim, sobre a sífilis congênita, em se tratando de doença prevenível, facilmente identificável, com tratamento disponível no serviço público de saúde, considera-se a ocorrência identificada elevada (1,4%), sendo especialmente preocupante pelo aumento estatisticamente significativo entre os óbitos fetais tardios. Dados brasileiros evidenciam que as taxas de detecção de gestantes com sífilis têm mantido crescimento no período entre 2011-2022, porém com menor intensidade a partir de 2018: entre 2011 e 2017 o crescimento médio foi de 17,6%, seguida de estabilidade nos anos subsequentes e aumento de 16,7% em 2021^(29)^.

Estudo nacional apontou que dos 558 registros de óbitos no conjunto: sífilis, toxoplasmose, rubéola, citomegalovírus e vírus herpes simplex, 98,7% decorreram da sífilis congênita. Merece destaque o fato de a sífilis ser uma doença cujo diagnóstico e tratamento são de baixo custo e fácil acesso, que quando ofertados em tempo oportuno, pode levar à remissão completa da doença na mãe, impedindo que ocorra a transmissão vertical e evitando a possibilidade da ocorrência dos óbitos fetais por esta condição^(17)^. Diante disso, a sífilis constitui indicador de qualidade da assistência pré-natal, sendo visto como inaceitável o óbito relacionado a esta doença até mesmo nos sistemas de saúde mais precários, sugerindo deficiência na qualidade da assistência pré-natal ofertada às gestantes, problemas de cobertura na oferta do diagnóstico ou mesmo adesão deficitária, ou inexistente, ao tratamento para a sífilis^(30)^.

Constitui fragilidade do presente estudo o fato de tratarem-se de dados secundários obtidos exclusivamente do SIM e, como tal, não houve possibilidade de controlar diretamente sua qualidade, sendo especialmente relevante possível viés referente a causa básica do óbito por subnotificação ou erro de codificação na declaração de óbito, bem como por registro incompleto do diagnóstico. Também não foi possível avaliar a causalidade relativa aos óbitos, devido ao desenho observacional adotado, tampouco a avaliação de variáveis clínicas, maternas e fetais não registradas na declaração de óbito e o impacto de intervenções de saúde pública ocorridas no período do estudo.

Por outro lado, o uso de dados secundários permitiu a abordagem de dados do Brasil em uma década, situação especialmente relevante pelo fato do país apresentar dimensões continentais, bem como a possibilidade de estratificação da mortalidade fetal segundo a idade gestacional e a causa básica do óbito, aspectos não abordados em estudos nacionais anteriores.

A grande maioria dos óbitos fetais ocorreram por causas potencialmente preveníveis, principalmente a partir do acompanhamento perinatal, incluindo o diagnóstico e o tratamento precoces de doenças maternas e fetais. Assim, espera-se que este estudo subsidie gestores e profissionais da área da saúde que atendam ao grupo materno-infantil, em particular da área de enfermagem, nas especialidades obstétrica, neonatal e saúde da família, pois sua atuação pode impactar diretamente nessa realidade a partir do desenvolvimento de ações de educação em saúde, acompanhamento pré-natal sistemático, identificação precoce de sinais de risco materno-fetal, implementação de protocolos para o manejo de complicações durante o trabalho de parto e fortalecimento da vigilância epidemiológica nos diferentes pontos da Rede de Atenção à Saúde.

Para gestores, recomenda-se priorizar a qualificação da atenção pré-natal, garantindo oferta de consultas em número adequado, ultrassonografia de rotina e rastreamento das doenças maternas, como hipertensão e diabetes. É essencial fortalecer a rede de atenção perinatal, assegurando a vinculação precoce da gestante à maternidade de referência e a disponibilidade de protocolos clínicos atualizados para o manejo de complicações no parto e no nascimento. A ampliação de ações de educação permanente para equipes de saúde, o uso de sistemas de vigilância epidemiológica sensíveis, como a investigação oportuna de óbitos fetais e o monitoramento de indicadores de qualidade do pré-natal e da assistência ao parto podem ser incorporados como metas de gestão. Tais medidas podem reduzir iniquidades regionais, melhorar a coordenação da rede de atenção materno-infantil e impactar de forma significativa a redução da mortalidade fetal.

## CONCLUSÃO

A maior parte dos óbitos fetais ocorridos entre os anos de 2011 e 2020 está inserido no Capítulo 16 da CID-10 (algumas afecções originadas no período perinatal), destacando-se os grupos: feto e recém-nascido afetado por fatores maternos e complicações da gravidez, trabalho de parto e parto; hipóxia intrauterina e asfixia ao nascer e outras afecções originadas no período perinatal. Hipóxia e asfixia compõem a única categoria com tendência de diminuição para todos os tipos de óbito (precoce, intermediário ou tardio). Contudo, houve tendência de aumento das causas básicas: Anomalias cromossômicas não classificadas em outra parte e Feto e recém-nascido afetado por fatores maternos e por complicações da gravidez, trabalho de parto e parto, também para os óbitos precoce, intermediário ou tardio.

Destaca-se que a grande maioria das causas dos óbitos identificadas neste estudo são caracterizadas como evitáveis por uma adequada atenção ao pré-natal, parto e recém-nascido, o que evidencia a necessidade de qualificar o cuidado perinatal, visando diminuir e/ou evitar sua ocorrência. Esses resultados podem subsidiar políticas públicas de saúde voltadas para a prevenção de óbitos fetais, com foco especial em condições que ainda apresentam tendência de aumento.

## Data Availability

Todo o conjunto de dados que dá suporte aos resultados deste estudo está disponível mediante solicitação ao autor correspondente.

# Translation: Comportamiento temporal de la mortalidad proporcional de las muertes fetales según la causa básica, 2011–2020

**DOI:** 10.1590/1980-220X-REEUSP-2025-0288es

**Authors:** Júlia Rodrigues Soares de Barros, Ana Beatriz Henrique Parenti, Ana Paula Pinho Carvalheira, Anna Paula Ferrari, Marli Teresinha Cassamassimo Duarte, Cristina Maria Garcia de Lima Parada

**Affiliations:** 1 Universidade Estadual Paulista “Júlio de Mesquita Filho”, Faculdade de Medicina de Botucatu, Departamento de Enfermagem, Botucatu, SP, Brasil.

**Keywords:** Muerte Fetal, Epidemiologia, Factores de Riesgo, Sistemas de Información en Salud

## Abstract

**Objetivo:**

Investigar el comportamiento temporal de la mortalidad proporcional de las muertes fetales tempranas, intermedias y tardías entre 2011 y 2020, según la causa básica.

**Métodos:**

Estudio de base poblacional realizado con datos secundarios de muertes fetales registrados en el Sistema de Información de Mortalidad correspondientes al periodo comprendido entre 2011 y 2020.

**Resultados:**

Las mayores medias anuales de mortalidad proporcional según la causa básica de las muertes fetales tempranas, intermedias o tardías fueron, respectivamente: feto y recién nacido afectados por factores maternos y complicaciones del embarazo, el trabajo de parto y el parto (41.3%, 44.4% y 44.0%); hipoxia intrauterina y asfixia al nacer (22.3%, 21.6% y 22.8%) y otras afecciones originadas en el periodo perinatal (28.0%, 23.4% y 24.4%). La hipoxia y la asfixia mostraron una tendencia a la disminución en todos los tipos de muerte. El grupo de fetos y recién nacidos afectados por factores maternos y por complicaciones del embarazo, el trabajo de parto y el parto mostró una tendencia al alza.

**Conclusión:**

Las principales causas básicas de muerte se caracterizan por ser evitables mediante una atención adecuada durante el prenatal, el parto y el posparto, lo que pone de manifiesto la necesidad de mejorar la atención perinatal con el fin de reducir y/o evitar su ocurrencia.

## INTRODUCCIÓN

La Organización Mundial de la Salud (OMS) y la Clasificación Estadística Internacional de Enfermedades y Problemas Relacionados con la Salud - 11.ª revisión (CID-11) definen la muerte fetal como la muerte de un feto antes de su expulsión o extracción completa del cuerpo de una mujer, independientemente de la duración del embarazo^(1)^. Constituye un problema significativo de salud pública mundial y un indicador importante de la salud materno-infantil^(2)^, ya que refleja la calidad de la asistencia y las condiciones de acceso a la atención médica disponible para las embarazadas desde la etapa prenatal hasta el momento del parto^(3,4)^.

La muerte fetal se puede clasificar como precoz (menos de 20 semanas de gestación), intermedia (entre 20 y 28 semanas) y tardía (después de 28 semanas), o bien como anteparto o intraparto, según el período en que se produce^(5)^, siendo este desenlace negativo más frecuente en países de ingresos medios y bajos^(6)^. En países de ingresos altos, como los Estados Unidos de América, la mejora de la atención a las embarazadas en el último siglo ha dado lugar a una importante reducción de las muertes fetales y a una disminución proporcionalmente mayor de las muertes neonatales, de modo que la muerte fetal sigue siendo un problema significativo y poco estudiado, responsable de casi el 50% de todas las muertes perinatales en el país^(7)^. En Brasil, la tasa de mortalidad fetal global en el período comprendido entre 1996 y 2021 fue de 11,4/1000 nacidos vivos, con una tendencia a la baja en todas las regiones, con algunas fluctuaciones puntuales, siendo la caída más pronunciada la registrada en la región sudeste, con una reducción del 48%^(8)^.

La muerte fetal puede verse influida por la concurrencia simultánea de diversos factores, y este carácter multifactorial puede dificultar la detección de la causa específica^(9)^. Entre los factores asociados a resultados perinatales adversos, incluida la muerte fetal, se encuentran: edad materna superior a 35 años; nuliparidad; tabaquismo; pérdidas fetales anteriores; enfermedades maternas previas, como hipertensión arterial, diabetes mellitus y obesidad; infecciones maternas durante el embarazo; complicaciones placentarias, umbilicales, amnióticas o uterinas; anomalías congénitas; asfixia y traumatismos al nacer; restricción del crecimiento fetal y ausencia o mala calidad de la atención prenatal. Además, algunos factores sociales, como el bajo nivel educativo, el color de piel negro o moreno de la madre y las condiciones socioeconómicas precarias, también se asocian con las muertes fetales^(10)^.

Un estudio nacional sobre las muertes fetales ocurridas entre 1996 y 2021^(8)^ las presentó a partir de la tasa de mortalidad global, sin tener en cuenta la clasificación según la edad gestacional en que se produjeron, una laguna que se pretende abordar en el presente estudio, cuyo objetivo es investigar la evolución temporal de la mortalidad proporcional de las muertes fetales tempranas, intermedias y tardías entre 2011 y 2020, según su causa básica y la macrorregión en que se produjeron, con el fin de apoyar acciones para mejorar la salud materno-infantil y la adopción de medidas preventivas que eviten su ocurrencia.

## MÉTODO

### Diseño del Estudio

Se trata de un estudio observacional, retrospectivo, de base poblacional, realizado a partir de datos secundarios del Sistema de Información sobre Mortalidad (SIM) sobre muertes fetales. En su elaboración se utilizó la Declaración STROBE^(11)^.

### Población y Fuente de Datos

Se incluyeron en el estudio todos los casos de muerte fetal ocurridos en Brasil y registrados en el SIM en el periodo de 2011 a 2020. El SIM es un sistema nacional de vigilancia epidemiológica cuyo objetivo es recopilar datos sobre las muertes ocurridas en el país, siendo su documento básico la Declaración de Defunción (DD), cuyo uso es obligatorio, con el fin de que las estadísticas de mortalidad producidas sean válidas y comparables. El sistema presenta una completitud clasificada como excelente (menos del 5% de incompletitud) y, a pesar de las disparidades en la calidad de sus diferentes dimensiones, los estudios indican mejoras a lo largo del tiempo en los registros de datos de mortalidad de la población brasileña^(12)^.

### Variables del Estudio

Se utilizaron variables relacionadas con las características maternas y fetales, datos del embarazo y el parto, y relativos a la muerte: Maternas: edad (años completos); nivel educativo (hasta 3, 4–7, 8–11, 12 o más); actividad remunerada (sí, no); número de hijos vivos (0–3, ≥4); hijos fallecidos (sí, no); región de residencia (Norte, Noreste, Centro-Oeste, Sudeste, Sur). Fetales: sexo (masculino, femenino); peso al nacer (<1500, 1500–2499, ≥2500); raza/color de piel (blanca, morena, negra, amarilla, indígena). Datos sobre el embarazo y el parto: tipo de embarazo (único, múltiple); tipo de parto (vaginal, cesárea); edad gestacional (semanas completas). Datos relativos a la muerte: trimestre en que se produjo (primero, segundo y tercero); momento de la muerte (antes del parto, después del parto); macrorregión en que se produjo (norte, noreste, centro-oeste, sureste, sur); asistencia médica en el momento de la muerte (sí, no). Causa básica por grupo, según el capítulo de la CIE-10^(13)^: Capítulos: I - enfermedades infecciosas y parasitarias; II - neoplasias/tumores; III - enfermedades de la sangre, de los órganos hematopoyéticos y algunos trastornos inmunitarios; IV - enfermedades endocrinas, nutricionales y metabólicas; V - trastornos mentales y del comportamiento; VI - enfermedades del sistema nervioso; VII - enfermedades del ojo y sus anexos; VIII - enfermedades del oído y de la apófisis mastoides; IX - enfermedades del aparato circulatorio; X - enfermedades del aparato respiratorio; XI - enfermedades del aparato digestivo; XII - enfermedades de la piel y del tejido subcutáneo; XIII - enfermedades del sistema osteomuscular y del tejido conjuntivo; XIV - enfermedades del aparato genitourinario; XV - embarazo, parto y puerperio; XVI - algunas afecciones originadas en el período perinatal; XVII - malformaciones congénitas, deformidades y anomalías cromosómicas; XVIII - síntomas, signos y hallazgos anormales de exámenes clínicos y de laboratorio, no clasificados en otra parte; XIX - lesiones, envenenamientos y algunas otras consecuencias de causas externas; XX - causas externas de morbilidad y mortalidad; XXI - factores que influyen en el estado de salud y el contacto con los servicios de salud; XXII - códigos para fines especiales.

### Métodos Estadísticos

Inicialmente, se realizó un análisis descriptivo de las variables relativas a las características maternas y fetales, el embarazo, el parto y la muerte. Para investigar la existencia de una tendencia al aumento o la disminución de la mortalidad proporcional de las muertes fetales por causa básica y macrorregión de ocurrencia, en las poblaciones de muerte fetal temprana, intermedia y tardía, se ajustaron modelos de regresión lineal con respuesta normal para cada serie temporal de mortalidad proporcional, considerando el tiempo como variable independiente. Además, la tendencia de cada serie se comprobó mediante la prueba de Cox-Stuart. Los análisis se realizaron con el software SPSS 21.

### Aspectos Éticos

Se garantizó la preservación de los aspectos éticos, de acuerdo con la Resolución del Consejo Nacional de Salud n.º 510, de 7 de abril de 2016, párrafo único, que exime del registro y la evaluación por parte del sistema Comité de Ética en Investigación/Comisión Nacional de Ética en Investigación (CEP/CONEP) a las investigaciones que utilicen información de acceso público^(14)^.

## RESULTADOS

En la década 2011–2020 se registraron 164 504 muertes fetales en el SIM. El capítulo 16 de la CIE-10 (algunas afecciones originadas en el período perinatal) incluyó la mayor parte de ellas, seguido del capítulo 17 (malformaciones congénitas, deformidades y anomalías cromosómicas) y el capítulo 1 (algunas enfermedades infecciosas y parasitarias), como se muestra en la Tabla 1.

**Tabla 1:** Descripción de las muertes por capítulo de la CIE-10 – Brasil, 2011–2020.

Capítulo	Categoría	n	%
**1 Enfermedades infecciosas y parasitarias**				
Sífilis congénita		2340	1,4
**16 Algunas afecciones originadas en el período perinatal**			
Feto y recién nacido afectados por factores maternos y por complicaciones del embarazo, el trabajo de parto y el alumbramiento		72436	44,0
Otras afecciones originadas en el período perinatal		40138	24,4
Hipoxia intrauterina y asfixia al nacer		36891	22,4
Restricción del crecimiento fetal, desnutrición fetal y trastornos relacionados con la gestación corta y el bajo peso al nacer		1844	1,1
Enfermedad hemolítica del feto y del recién nacido		266	0,2
Otras infecciones específicas del período perinatal		277	0,2
Enfermedades infecciosas y parasitarias congénitas		175	0,1
Traumatismos durante el parto		98	0,06
Otros trastornos respiratorios originados en el período perinatal		48	0,03
**17 Malformaciones congénitas, deformidades y anomalías cromosómicas**		3997	2,4
Otras malformaciones congénitas			
Otras malformaciones congénitas del sistema nervioso		2409	1,5
Anomalías cromosómicas, no clasificadas en otra parte		1049	0,7
Malformaciones congénitas del aparato circulatorio		1247	0,7
Otras malformaciones y deformidades congénitas del aparato osteomuscular		662	0,4
Otras malformaciones del aparato genitourinario		377	0,2
Otras malformaciones congénitas del aparato digestivo		129	0,08
Espina bífida		74	0,04
Labio leporino y paladar hendido		31	0,02
Ausencia, atresia y estenosis del intestino delgado		16	0,01

En relación con los promedios anuales de mortalidad proporcional, según la causa básica, destacaron las siguientes categorías, considerando la ocurrencia de muerte fetal temprana, intermedia o tardía, respectivamente: feto y recién nacido afectados por factores maternos y complicaciones del embarazo, trabajo de parto y parto (41.3%, 44.4% y 44.0%); hipoxia intrauterina y asfixia al nacer (22.3%, 21.6% y 22.8%) y otras afecciones originadas en el período perinatal (28.0%, 23.4% y 24.4%), todas del Capítulo 16 de la CIE 10 (datos no presentados en la tabla).

La hipoxia intrauterina y la asfixia al nacer mostraron una tendencia a la disminución en todos los tipos de muerte: precoz, intermedia y tardía. También se observó una tendencia a la disminución de otras infecciones específicas del período perinatal en los casos de muertes fetales intermedias y otras malformaciones del sistema nervioso, entre las muertes fetales tardías (Tabla 2).

**Tabla 2:** Investigación de la tendencia de la mortalidad fetal proporcional según la causa básica de muerte – Brasil, 2011–2020.

Categoría	Precoz			Intermedia			Tardía		
	β (IC95%)	*p*	Tendencia	β (IC95%)	*p*	Tendencia	β (IC95%)	*p*	Tendencia
Anomalías cromosómicas, no clasificadas en otra parte	0,05 (0,01–0,09)	*0,019*	Aumento	0,05 (0,03–0,08)	*0,001*	Aumento	0,06 (0,04– 0,07)	*<0,001*	Aumento
Ausencia, atresia y estenosis del intestino delgado	0,00 (–0,01–0,01)	*0,873*	Estacionario	0,00 (0,00–0,00)	*0,244*	Estacionario	0,00 (0,00– 0,00)	*0,700*	Estacionario
Enfermedad hemolítica del feto y del recién nacido	0,00 (–0,02–0,02)	*0,752*	Estacionario	0,01 (–0,01–0,02)	*0,223*	Estacionario	0,00 (–0,01– 0,00)	*0,194*	Estacionario
Enfermedades infecciosas y parasitarias congénitas	–0,02 (–0,04–0,00)	*0,074*	Estacionario	0,00 (–0,01–0,01)	*0,778*	Estacionario	0,00 (0,00–0,01)	*0,168*	Estacionario
Espina bífida	0,01 (–0,01–0,02)	*0,388*	Estacionario	0,01 (–0,01–0,02)	*0,287*	Estacionario	0,00 (–0,01–0,00)	*0,274*	Estacionario
Labio leporino y paladar hendido	0,00 (–0,01–0,00)	*0,122*	Estacionario	0,00 (0,00–0,00)	*0,997*	Estacionario	0,00 (0,00–0,00)	*0,491*	Estacionario
Feto y recién nacido afectados por factores maternos y por complicaciones del embarazo, el parto y el alumbramiento	0,74 (0,26–1,21)	*0,007*	Aumento	0,53 (0,27–0,78)	*0,001*	Aumento	0,53 (0,28–0,79)	*0,001*	Aumento
Hipoxia intrauterina y asfixia al nacer	–0,44 (–0,81 – –0,07)	*0,024*	Disminución	–0,48 (–0,67 – –0,28)	*<0,001*	Disminución	–0,55 (–0,84 – –0,26)	*0,002*	Disminución
Malformaciones congénitas del aparato circulatorio	0,01 (–0,04–0,06)	*0,615*	Estacionario	0,05 (0,02–0,07)	*0,004*	Aumento	0,04 (0,02–0,07)	*0,002*	Aumento
Otras afecciones originadas en el período perinatal	–0,31 (–0,92–0,30)	*0,273*	Estacionario	–0,19 (–0,47–0,09)	*0,151*	Estacionario	–0,19 (–0,67–0,29)	*0,394*	Estacionario
Otras infecciones específicas del período perinatal	0,02 (–0,02–0,05)	*0,340*	Estacionario	–0,02 (–0,03 – –0,01)	*0,002*	Disminución	–0,01 (–0,02 – 0,00)	*0,077*	Estable
Otras malformaciones congénitas	–0,03 (–0,11–0,05)	*0,360*	Estacionario	0,04 (–0,06–0,13)	*0,401*	Estacionario	0,00 (–0,04–0,03)	*0,889*	Estacionario
Otras malformaciones congénitas del aparato digestivo	0,00 (–0,01–0,01)	*0,737*	Estacionario	0,00 (–0,01–0,01)	*0,550*	Estacionario	0,00 (–0,01–0,00)	*0,035*	Irrelevante
Otras malformaciones congénitas del sistema nervioso	0,05 (–0,01–0,11)	*0,107*	Estacionario	0,02 (–0,06–0,09)	*0,613*	Estacionario	–0,05 (–0,08 – –0,03)	*0,002*	Disminución
Otras malformaciones del aparato genitourinario	0,02 (–0,02–0,07)	*0,278*	Estacionario	0,00 (–0,03–0,03)	*0,913*	Estacionario	0,01 (0,00–0,03)	*0,089*	Estacionario
Otras malformaciones y deformidades congénitas del aparato osteomuscular	0,03 (0,00–0,06)	*0,038*	Aumento	0,01 (–0,01–0,02)	*0,535*	Estacionario	0,02 (0,00–0,03)	*0,027*	Aumento
Otros trastornos respiratorios originados en el período perinatal	0,00 (–0,01–0,01)	*0,742*	Estacionario	0,00 (–0,01–0,01)	*0,633*	Estacionario	0,00 (–0,01–0,00)	*0,794*	Estacionario
Retraso en el crecimiento fetal, desnutrición fetal y trastornos relacionados con la gestación corta y el bajo peso al nacer	–0,15 (–0,36– 0,06)	*0,127*	Estacionario	–0,04 (–0,18–0,09)	*0,479*	Estacionario	0,03 (0,00–0,06)	*0,085*	Estacionario
Sífilis congénita	0,03 (–0,03–0,08)	*0,345*	Estacionario	0,04 (–0,06–0,15)	*0,400*	Estacionario	0,13 (0,06–0,19)	*0,003*	Aumento
Traumatismo durante el parto	0,00 (–0,01–0,02)	*0,721*	Estacionario	–0,01 (–0,02–0,00)	*0,119*	Estacionario	0,00 (–0,01–0,01)	*0,295*	Estacionario

Las categorías que presentaron un aumento estadísticamente significativo en la tendencia para todos los tipos de muerte fueron: anomalías cromosómicas no clasificadas en otra parte y feto y recién nacido afectados por factores maternos y complicaciones del embarazo, el trabajo de parto y el parto. Hubo una tendencia al alza en el caso de las muertes fetales tempranas por otras malformaciones y deformidades congénitas osteomusculares. En el caso de las muertes intermedias y tardías, se observó una tendencia al alza en las malformaciones congénitas del aparato circulatorio y, solo entre las muertes tardías, se observó una tendencia al alza en otras malformaciones y deformidades congénitas del aparato osteomuscular y la sífilis congénita (Tabla 2).

Entre las muertes fetales tempranas, las series temporales de mortalidad proporcional según las categorías: anomalías cromosómicas no clasificadas en otra parte; feto y recién nacido afectados por factores maternos y por complicaciones del embarazo, trabajo de parto y parto, y otras malformaciones y deformidades congénitas osteomusculares mostraron una tendencia al alza anual, mientras que la serie de mortalidad proporcional según la categoría de hipoxia intrauterina y asfixia al nacer mostró una tendencia a la baja anual (Figura 1).

**Figura 1:** Análisis de la tendencia anual de las muertes fetales tempranas según la macrorregión de ocurrencia y la categoría de la CIE-10.

En lo que respecta a la mortalidad proporcional de las muertes fetales tempranas, según las macrorregiones, se observó un aumento en las categorías: anomalías cromosómicas en la región sur (β = 0,23, IC 95% = 0,04–0,41, p = 0,021); fetos y recién nacidos afectados por factores maternos y por complicaciones del embarazo, el trabajo de parto y el parto en la región norte (β = 2,10, IC95% = 0,82–3,38, p = 0,005) y una tendencia a la disminución en la categoría de hipoxia intrauterina y asfixia al nacer en las regiones centro oeste y sur, respectivamente (β = –1,05, IC95% = –1,84 – –0,25, p = 0,017) y (β = –1,00, IC95% = –1,89 – –0,11, p = 0,032). No hubo resultados significativos en la categoría de otras malformaciones y deformidades congénitas del aparato osteomuscular por macrorregión (Figura 1).

Entre las muertes fetales intermedias, las series temporales de mortalidad proporcional según las categorías: anomalías cromosómicas no clasificadas en otra parte; fetos y recién nacidos afectados por factores maternos y por complicaciones del embarazo, el parto y el alumbramiento, y malformaciones congénitas del aparato circulatorio experimentaron un aumento, mientras que las categorías de hipoxia intrauterina y asfixia al nacer y otras infecciones específicas del período perinatal disminuyeron en el mismo período (Figura 2).

**Figura 2:** Análisis de la tendencia anual de las muertes fetales intermedias según la macrorregión de ocurrencia y la categoría de la CIE-10.

En cuanto a la mortalidad proporcional de las muertes fetales intermedias, según las macrorregiones, se observó un aumento en las categorías: anomalías cromosómicas en las regiones sur y centro-oeste (β = 0,20, IC95% = 0,08–0,31, p = 0,004) y (β = 0,11, IC95% = 0,004 –0,18, p = 0,006), respectivamente; fetos y recién nacidos afectados por factores maternos y por complicaciones del embarazo, el parto y el alumbramiento en las regiones norte (β = 2,88, IC95% = 2,34–3,42, p < 0,001) y noreste (β = 0,50, IC95% = 0,21-0,79, p = 0,004) y sífilis congénita en la región centro-oeste (β = 0,17, IC95% = 0,08–0,27, p = 0,002). Se observó una tendencia a la disminución en la categoría de hipoxia intrauterina y asfixia al nacer en las regiones noreste, centro-oeste y sur (β = –0,77, IC del 95% = –1,21– –0,33, p = 0,004), (β = –0,95, IC del 95% = –1,52 – –0,38, p = 0,005) y (β= –0,67, IC95%= –1,08 – –0,25, p = 0,006), respectivamente. No se observaron resultados significativos en la categoría «otras infecciones específicas del periodo perinatal y malformaciones congénitas del aparato circulatorio» cuando se consideró la macrorregión de ocurrencia (Figura 2).

Entre las muertes fetales tardías, las series temporales de mortalidad proporcional según las categorías: anomalías cromosómicas no clasificadas en otra parte; feto y recién nacido afectados por factores maternos y por complicaciones del embarazo, el parto y el alumbramiento; malformaciones congénitas del aparato circulatorio; otras malformaciones y deformidades congénitas del aparato osteomuscular y sífilis congénita de la CIE-10 mostraron una tendencia al alza anual, mientras que la serie de mortalidad proporcional según la categoría: hipoxia intrauterina y asfixia al nacer y otras malformaciones congénitas del sistema nervioso mostró una tendencia a la baja anual (Figura 3).

**Figura 3:** Análisis de la tendencia anual de las muertes fetales tardías según la macrorregión de ocurrencia y la categoría de la CIE-10.

En cuanto a la mortalidad proporcional de las muertes fetales tardías, según las macrorregiones, se observó un aumento en las categorías de anomalías cromosómicas en las regiones noreste (β = 0,02, IC95% = 0,00–0,04, p = 0,036, centro-oeste (β = 0,10, IC95% = 0,04–0,17, p = 0,0050, sureste (β = 0,06, IC95% = 0,02–0,11, p = 0,010) y sur (β = 0,17, IC95% = 0,10–0,23, p < 0,001); fetos y recién afectado por factores maternos y por complicaciones del embarazo, el parto y el alumbramiento en las regiones norte (β = 2,35, IC95% = 1,83–2,87, p < 0,001) y noreste (β = 0,42, IC95% = 0,00–0,84, p = 0,005); malformación del aparato circulatorio en las regiones norte (β = 0,06, IC95% = 0,02–0,10, p = 0,005), noreste (β = 0,06, IC95% = 0,01–0,11, p = 0,035) y sureste (β = 0,03, IC95% = 0.00–0.06, p = 0.027); malformación del sistema nervioso en las regiones noreste (β = –0.07, IC95% = –0.11 – –0.03, p = 0.002) y sur (β= –0.14, IC95% = –0.22 – –0,06, p = 0,004); malformaciones osteomusculares en las regiones norte (β = 0,06, IC 95% = 0,03–0,09, p = 0,002) y sur (β = 0,06, IC95% = 0,01–0,10, p = 0,014) y sífilis en las regiones centro-oeste (β = 0,25, IC95% = 0,18–0,32, p < 0,001) y sureste (β = 0,20, IC95% = 0,11–0,29, p = 0,001). Se observó una tendencia a la disminución en la categoría de hipoxia intrauterina y asfixia al nacer en las regiones noreste (β = –0,82, IC95% = –1,18 – –0,46, p = 0,001), centro-oeste (β = –0,79, IC95% = –1,10 – –0,47, p < 0,001) y sur (β = –0,70, IC95%= –0,97 – –0,43, p < 0,001) (Figura 3)

## DISCUSIÓN

Este estudio permitió conocer la evolución temporal de la mortalidad proporcional de las muertes fetales según la causa básica en el periodo de 2011 a 2020, siendo que el capítulo 16 de la CIE-10 (algunas afecciones originadas en el periodo perinatal) incluyó la mayor parte de las muertes fetales ocurridas: 92,5%, seguido del capítulo 17 (malformaciones congénitas, deformidades y anomalías cromosómicas): 6,1% y el capítulo 1 (enfermedades infecciosas y parasitarias): 1,4%.

Por lo expuesto, fue posible identificar una tendencia al alza en la causa básica de fetos y recién nacidos afectados por factores maternos y por complicaciones del embarazo, el trabajo de parto y el parto en las regiones Norte y Noreste y, en cuanto a las demás causas, no fue posible identificar un patrón de aumento o disminución en la tendencia de la mortalidad fetal proporcional, según la causa básica de muerte, de acuerdo con las diferentes regiones del país.

Un estudio que analizó las muertes fetales ocurridas en la región centro-oeste entre 2008 y 2018 mostró que el 93% de las muertes incluidas en el estudio estaban asociadas al capítulo 16 de la CIE-10 (algunas afecciones originadas en el período perinatal), un hallazgo similar al del presente estudio, en el que el 92,5% de las muertes también se asociaron al capítulo 16. Cabe destacar que la gran mayoría de las afecciones originadas en el período perinatal son susceptibles de prevención, lo que sugiere la necesidad de mejorar la calidad de la atención prestada, tanto en el período prenatal como en el parto^(15,16)^.

La asfixia perinatal, que incluye la hipoxia intrauterina y la asfixia al nacer, mostró una tendencia a la disminución en todos los tipos de muertes. Un estudio que analizó la epidemiología de la mortalidad perinatal, según la clasificación de Wigglesworth, destacó que, del 58,9% de las muertes fetales, solo el 0,8% tuvo como causa la asfixia^(17)^. La relevancia de esta categoría en el presente estudio se debe a la alta frecuencia identificada en el periodo, ya que aproximadamente el 20% de las muertes tuvieron esta causa básica. Ante esto, el resultado de la tendencia a la disminución de la ocurrencia de muertes fetales tempranas, intermedias y tardías relacionadas con esta causa indica que la calidad de la atención prestada, tanto a la embarazada como al feto, puede haber mejorado a lo largo del periodo estudiado. Un estudio cuyo objetivo era analizar los indicadores relacionados con la atención prenatal, utilizando la información de las ediciones de la Encuesta Nacional de Salud de 2013 y 2019, encontró un aumento significativo en el número de consultas prenatales y exámenes clínicos y obstétricos, en la proporción de mujeres que recibieron orientación sobre el servicio de referencia para el parto y en la realización de pruebas de sífilis y VIH. Los autores concluyeron que se han producido avances importantes en la cobertura y la calidad de la atención a las embarazadas en Brasil, aunque persisten retos como el inicio tardío de la atención prenatal y la orientación aún insuficiente sobre los servicios de referencia para el parto^(18)^.

La disminución de la incidencia de muertes fetales por malformaciones del sistema nervioso puede deberse a la introducción de la suplementación con ácido fólico en el período periconcepcional y durante el primer trimestre de gestación, lo que ha dado lugar a una disminución de aproximadamente el 50% de los defectos del tubo neural, así como al diagnóstico precoz de malformaciones a partir de exámenes de imagen realizados durante la gestación y nuevas técnicas neuroquirúrgicas intrauterinas, lo que ha mejorado el resultado perinatal^(19)^.

En cuanto a las anomalías cromosómicas no clasificadas en otra parte, un estudio realizado en la Guayana Francesa destacó que el 6% de las muertes se debieron a anomalías fetales, de las cuales casi el 50% fueron consecuencia de anomalías cromosómicas^(20)^, y un estudio realizado en el noreste de Brasil evidenció que, aunque el 2,9% de las muertes fetales se debieron a malformaciones congénitas, se produjo un aumento estadísticamente significativo de estos casos^(21)^. Un estudio ecológico de base poblacional realizado en Brasil comparó las malformaciones que se produjeron entre mujeres de entre 35 y 40 años y mayores de 41 años y, contrariamente a lo esperado, cuando se analizaron las malformaciones por subgrupo de edad, se observó una tendencia al alza solo entre las más jóvenes, destacando los autores que este hallazgo puede deberse al pequeño tamaño del subgrupo de mayor edad^(22)^.

Dado que hay pruebas de la asociación entre las malformaciones y la edad materna avanzada, uno de los factores que puede estar relacionado con el mencionado aumento es la opción de las mujeres de posponer el embarazo, como consecuencia del proceso de urbanización y de su interés por la escolarización, factores relacionados con la caída de la tasa de fecundidad y el proceso de transición demográfica por el que atraviesa Brasil^(23)^. Además, se debe considerar la posibilidad de que el aumento en la incidencia esté relacionado con la mejora diagnóstica por el aumento en la disponibilidad del uso de la ecografía obstétrica. Desde su introducción en la medicina, esta tecnología ha evolucionado significativamente, generando imágenes cada vez más precisas y detalladas, lo que permite el seguimiento del desarrollo fetal y la detección de malformaciones, con la posibilidad de detectar el síndrome de Down y grandes anomalías estructurales ya en el primer trimestre, y otras anomalías fetales graves a partir del segundo trimestre^(24)^.

En cuanto a la categoría de fetos y recién nacidos afectados por factores maternos y por complicaciones del embarazo, el trabajo de parto y el parto, un estudio internacional señaló que las condiciones maternas fueron responsables del 24,2% de las muertes fetales, ocupando el segundo lugar entre las principales causas de estas muertes. Entre estas condiciones, la principal incluida fue la enfermedad hipertensiva del embarazo (82,5%) y los autores relacionaron la predominancia de esta causa con la composición étnica de la población, mayoritariamente afrocaribeña, además del bajo nivel socioeconómico. La primera causa asociada a la ocurrencia de mortinatos fueron las complicaciones obstétricas, más específicamente, la asfixia asociada al trabajo de parto (57%) y el desprendimiento prematuro de placenta (26%), que en este estudio se asoció a trastornos hipertensivos en el 47% de las pacientes^(20)^. Otro estudio realizado en la India también destacó que la condición materna más asociada a la ocurrencia de mortinatalidad fue la enfermedad hipertensiva del embarazo, que osciló entre el 6.4% y el 27.6%.

La mayoría de las condiciones maternas asociadas a la ocurrencia de estas muertes, así como las complicaciones del embarazo, el trabajo de parto y el parto, son modificables a partir del diagnóstico precoz y el tratamiento adecuado mediante una atención prenatal y un parto de calidad^(25)^. Ante esto, se destaca la relevancia de esta categoría, ya que aproximadamente el 40% de las muertes analizadas en la presente investigación se asociaron a ella, lo que sugiere la existencia de fallos en el seguimiento, el diagnóstico y el tratamiento precoz de problemas de salud materna pasados y actuales, complicaciones fetales de la placenta y anexos, complicaciones del trabajo de parto y complicaciones relacionadas con los hábitos maternos. El aumento de la tendencia de esta causa básica en las regiones Norte y Noreste puede justificarse, además de por las características de la población, por las desigualdades sociales a las que están sometidas, demostradas por la mayor proporción de mujeres que no acuden a ninguna consulta y la menor proporción de mujeres que acuden a siete o más consultas prenatales^(18).^

En cuanto a las malformaciones congénitas del aparato circulatorio, se observó un aumento entre las muertes intermedias y tardías. Estudios nacionales e internacionales han demostrado que, entre las muertes por malformaciones congénitas, las del aparato circulatorio eran las más frecuentes (35,3% y 32,0%), respectivamente^(26,27)^. Aunque la mayoría de los resultados de las anomalías congénitas son la muerte fetal, gran parte de ellas son potencialmente tratables si se realiza un diagnóstico precoz, lo que puede mejorar los resultados en las anomalías conocidas como no letales^(28)^.

En cuanto a las demás malformaciones y deformidades congénitas del aparato osteomuscular, con un aumento en el período de muertes fetales tempranas y tardías, el estudio describió que, de 46 705 nacidos vivos, 485 tenían malformaciones congénitas y, de estos, el 42,1% estaban relacionadas con el aparato osteomuscular, siendo esta la malformación congénita más predominante entre los nacidos vivos analizados en el estudio. Los autores destacan que esto puede deberse a que son afecciones visibles y fácilmente detectables en el examen físico, lo que da lugar a un diagnóstico precoz en el período posnatal inmediato. También destacan que, al influir de manera relevante en la tasa de mortalidad infantil, las malformaciones congénitas representan un problema de salud pública, por lo que requieren una atención adecuada y cualificada, con el fin de prevenir y reducir la morbilidad y la mortalidad^(26)^.

Por último, en cuanto a la sífilis congénita, al tratarse de una enfermedad prevenible, fácilmente identificable y con tratamiento disponible en el servicio público de salud, se considera que la incidencia identificada es elevada (1,4%), lo que resulta especialmente preocupante por el aumento estadísticamente significativo entre las muertes fetales tardías. Los datos brasileños muestran que las tasas de detección de embarazadas con sífilis han seguido aumentando en el periodo comprendido entre 2011 y 2022, aunque con menor intensidad a partir de 2018: entre 2011 y 2017, el crecimiento medio fue del 17,6%, seguido de una estabilidad en los años siguientes y un aumento del 16,7% en 2021^(29)^.

Un estudio nacional señaló que de los 558 registros de muertes en conjunto: sífilis, toxoplasmosis, rubéola, citomegalovirus y virus del herpes simple, el 98,7% se debieron a la sífilis congénita. Cabe destacar que la sífilis es una enfermedad cuyo diagnóstico y tratamiento son de bajo costo y fácil acceso, que cuando se ofrecen en el momento oportuno, pueden llevar a la remisión completa de la enfermedad en la madre, impidiendo la transmisión vertical y evitando la posibilidad de que se produzcan muertes fetales por esta condición^(17)^. Por lo tanto, la sífilis constituye un indicador de la calidad de la atención prenatal, y se considera inaceptable la muerte relacionada con esta enfermedad incluso en los sistemas de salud más precarios, lo que sugiere deficiencias en la calidad de la atención prenatal ofrecida a las embarazadas, problemas de cobertura en la oferta de diagnóstico o incluso una adhesión deficitaria, o inexistente, al tratamiento de la sífilis^(30)^.

Una debilidad del presente estudio es el hecho de que se trata de datos secundarios obtenidos exclusivamente del SIM y, como tal, no fue posible controlar directamente su calidad, siendo especialmente relevante el posible sesgo relativo a la causa básica de la muerte por subnotificación o error de codificación en la declaración de defunción, así como por el registro incompleto del diagnóstico. Tampoco fue posible evaluar la causalidad relativa a las muertes, debido al diseño observacional adoptado, ni evaluar las variables clínicas, maternas y fetales no registradas en la declaración de defunción y el impacto de las intervenciones de salud pública que tuvieron lugar durante el período del estudio.

Por otro lado, el uso de datos secundarios permitió abordar los datos de Brasil en una década, situación especialmente relevante debido a que el país tiene dimensiones continentales, así como la posibilidad de estratificar la mortalidad fetal según la edad gestacional y la causa básica de la muerte, aspectos que no se abordaron en estudios nacionales anteriores.

La gran mayoría de las muertes fetales se produjeron por causas potencialmente prevenibles, principalmente a partir del seguimiento perinatal, incluyendo el diagnóstico y el tratamiento precoz de enfermedades maternas y fetales. Por lo tanto, se espera que este estudio sirva de apoyo a los gestores y profesionales de la salud que atienden al grupo materno-infantil, en particular en el área de enfermería, en las especialidades de obstetricia, neonatología y salud familiar, ya que su actuación puede tener un impacto directo en esta realidad a partir del desarrollo de acciones de educación en salud, seguimiento prenatal sistemático, identificación temprana de signos de riesgo materno-fetal, implementación de protocolos para el manejo de complicaciones durante el parto y fortalecimiento de la vigilancia epidemiológica en los diferentes puntos de la Red de Atención a la Salud.

Se recomienda a los gestores que den prioridad a la cualificación de la atención prenatal, garantizando la oferta de un número adecuado de consultas, ecografías de rutina y detección de enfermedades maternas, como la hipertensión y la diabetes. Es esencial fortificar la red de atención perinatal, asegurando la vinculación temprana de la embarazada con la maternidad de referencia y la disponibilidad de protocolos clínicos actualizados para el manejo de complicaciones durante el parto y el nacimiento. La ampliación de las acciones de educación continua para los equipos de salud, el uso de sistemas de vigilancia epidemiológica sensibles, como la investigación oportuna de las muertes fetales y el monitoreo de los indicadores de calidad de la atención prenatal y del parto, pueden incorporarse como objetivos de gestión. Estas medidas pueden reducir las desigualdades regionales, mejorar la coordinación de la red de atención materno-infantil y tener un impacto significativo en la reducción de la mortalidad fetal.

## CONCLUSIÓN

La mayoría de las muertes fetales ocurridas entre los años 2011 y 2020 se incluyen en el capítulo 16 de la CIE-10 (algunas afecciones originadas en el período perinatal), destacando los grupos: feto y recién nacido afectados por factores maternos y complicaciones del embarazo, el trabajo de parto y el parto; hipoxia intrauterina y asfixia al nacer y otras afecciones originadas en el período perinatal. La hipoxia y la asfixia constituyen la única categoría con tendencia a disminuir para todos los tipos de muerte (precoz, intermedia o tardía). Sin embargo, se observó una tendencia al alza en las causas básicas: anomalías cromosómicas no clasificadas en otra parte y feto y recién nacido afectados por factores maternos y por complicaciones del embarazo, el trabajo de parto y el parto, también para las muertes tempranas, intermedias o tardías.

Cabe destacar que la gran mayoría de las causas de muerte identificadas en este estudio se caracterizan por ser evitables mediante una atención adecuada durante el embarazo, el parto y el posparto, lo que pone de manifiesto la necesidad de mejorar la atención perinatal con el fin de reducir y/o evitar su ocurrencia. Estos resultados pueden servir de base para políticas públicas de salud orientadas a la prevención de muertes fetales, con especial atención a las condiciones que aún presentan una tendencia al alza.

## Data Availability

El conjunto de datos que respaldan las conclusiones de este estudio está disponible previa solicitud al autor correspondiente.
